# Author Correction: Systematic dissection of tumor-normal single-cell ecosystems across a thousand tumors of 30 cancer types

**DOI:** 10.1038/s41467-025-58068-y

**Published:** 2025-03-21

**Authors:** Junho Kang, Jun Hyeong Lee, Hongui Cha, Jinhyeon An, Joonha Kwon, Seongwoo Lee, Seongryong Kim, Mert Yakup Baykan, So Yeon Kim, Dohyeon An, Ah-Young Kwon, Hee Jung An, Se-Hoon Lee, Jung Kyoon Choi, Jong-Eun Park

**Affiliations:** 1 Graduate School of Medical Science and Engineering, Korea Advanced Institute of Science and Technology, Daejeon, Republic of Korea; 2 Department of Bio and Brain Engineering, Korea Advanced Institute of Science and Technology, Daejeon, Republic of Korea; 3 Division of Hematology-Oncology, Department of Medicine, Samsung Medical Center, Sungkyunkwan University School of Medicine, Seoul, Republic of Korea; 4 Division of Cancer Data Science, National Cancer Center, Bioinformatics Branch, Goyang, Republic of Korea; 5 Department of Pathology, CHA Bundang Medical Center, CHA University, Seongnam-si, Republic of Korea; 6 Department of Health Sciences and Technology, Samsung Advanced Institute of Health Science and Technology, Sungkyunkwan University School of Medicine, Seoul, Republic of Korea; 7 Penta Medix Co., Ltd., Seongnam-si, Gyeonggi-do Republic of Korea; 8 Biomedical Research Center, Korea Advanced Institute of Science and Technology, Daejeon, Republic of Korea

**Keywords:** Cancer microenvironment, Tumour heterogeneity, Cancer genomics, Cancer therapy, Tumour immunology

Correction to: *Nature Communications* 10.1038/s41467-024-48310-4, published online 14 May 2024

In the version of the article initially published, there were numerical errors in the Supplementary Data where some overall survival times were missing and where reported progression-free survival times eclipsed overall survival time. The overall survival times have been updated and based on the durable clinical benefit criteria of immunotherapy (complete response, partial response or stable disease with PFS > 6 months or OS > 1 year), three patients with stable disease (SMC__Pat50, SMC__Pat118, and SMC__Pat239) have been reclassified as responders.

Figures 5 and 6, Supplementary Fig. 20, Supplementary Data 8 and Source Data 19b have now been updated. Despite this reclassification, there were no significant changes in the cell state terms in Figs. 5e, 6e and Supplementary Fig. 20a. For comparison, the original figures are available in the Supplementary Information accompanying this amendment. The changes are made in the HTML and PDF versions of the article.

## Supplementary information

Original Figs. 5, 6, Supplementary Fig. 20

Updated Supplementary Information

Updated Supplementary Data 8

## Source data

Updated Source Data

