# Supplementary material for: Systematic dissection of tumor-normal single-cell ecosystems across a thousand tumors of 30 cancer types
Source: Nat Commun. 2024 May 14;15:4067. doi: 10.1038/s41467-024-48310-4 (PMC11094150; doi:10.1038/s41467-024-48310-4)
Supplement: Supplementary file 3 — Description of Additional Supplementary Files [file 41467_2024_48310_MOESM3_ESM.pdf]

<u>Description of Additional Supplementary Files</u>

Supplementary Data Legends

Supplementary Data 1. scRNA-seq datasets included in this study.

Supplementary Data 2. NMF-derived cell states for each major cell type and gene signatures from previous studies.

Supplementary Data 3. Spatial transcriptome datasets included in this study.

Supplementary Data 4. Universal hallmark gene signatures for each cell type. The p-values were calculated using the two-sided t-test and adjusted with the Benjamini-Hochberg method.

Supplementary Data 5. scRNA-seq datasets of patients treated with immunotherapy.

Supplementary Data 6. Common gene signatures upregulated in immunotherapy responders or non-responders. The p-values were calculated using the two-sided t-test and adjusted with the Benjamini-Hochberg method.

Supplementary Data 7. TLS gene signature. The p-values were calculated using the two-sided Wald test and adjusted with Benjamini-Hochberg method using PyDESeq2.

Supplementary Data 8. Clinical characteristics of patients included in our LC cohort.
