# Supplementary material for: Systematic dissection of tumor-normal single-cell ecosystems across a thousand tumors of 30 cancer types
Source: Nat Commun. 2024 May 14;15:4067. doi: 10.1038/s41467-024-48310-4 (PMC11094150; doi:10.1038/s41467-024-48310-4)
Supplement: Supplementary file 12 — Reporting Summary [file 41467_2024_48310_MOESM12_ESM.pdf]

nature portfolio

Corresponding author(s): Se-Hoon Lee, Jung Kyoon Choi, Jong-Eun Park

Last updated by author(s): Apr 25, 2024

Reporting Summary

Nature Portfolio wishes to improve the reproducibility of the work that we publish. This form provides structure for consistency and transparency in reporting. For further information on Nature Portfolio policies, see our Editorial Policies and the Editorial Policy Checklist.

Statistics

For all statistical analyses, confirm that the following items are present in the figure legend, table legend, main text, or Methods section.

n/a	Confirmed	
☐	☒	The exact sample size (n) for each experimental group/condition, given as a discrete number and unit of measurement
☐	☒	A statement on whether measurements were taken from distinct samples or whether the same sample was measured repeatedly
☐	☒	The statistical test(s) used AND whether they are one- or two-sided<br>*Only common tests should be described solely by name; describe more complex techniques in the Methods section.*
☒	☐	A description of all covariates tested
☐	☒	A description of any assumptions or corrections, such as tests of normality and adjustment for multiple comparisons
☐	☒	A full description of the statistical parameters including central tendency (e.g. means) or other basic estimates (e.g. regression coefficient) AND variation (e.g. standard deviation) or associated estimates of uncertainty (e.g. confidence intervals)
☐	☒	For null hypothesis testing, the test statistic (e.g. F, t, r) with confidence intervals, effect sizes, degrees of freedom and P value noted<br>*Give P values as exact values whenever suitable.*
☒	☐	For Bayesian analysis, information on the choice of priors and Markov chain Monte Carlo settings
☒	☐	For hierarchical and complex designs, identification of the appropriate level for tests and full reporting of outcomes
☐	☒	Estimates of effect sizes (e.g. Cohen's d, Pearson's r), indicating how they were calculated

Our web collection on statistics for biologists contains articles on many of the points above.

Software and code

Policy information about availability of computer code

Data collection	No software was used for data collection
Data analysis	R packages: Seurat (v.4.1.1), Corto (v1.1.10), Survminer (v.0.4.2), WGCNA (v.1.71), circlize (v.0.4.15), Meta (v.1.1-2), RColorBrewer (v.1.1.3), ggplot2 (v.3.4.1), wesanderson (v.0.3.6), survival (v.3.1.12), ggpubr (v.0.4.0), data.table (v.1.14.4), dplyr (v.1.0.10)<br>Python packages: Scanpy (v.1.8.2), Scrublet (v.0.2.3), scikit-learn (v.1.0.2), BBKNN (v.1.5.1), gseapy (v.0.10.8), HTSeq (v.0.12.4), Geometric Sketch (v.1.2), infercnvpy (v. 0.4.2), statsmodels.stats (v.0.13.5), scipy.stats (v.1.10.0), Cell2location(v. 0.1.3), PyDESeq2 (v.0.4.3)<br>Others: Gephi (v.0.10.1), Trimmomatic (v.0.39), SortmeRNA (v.2.1b), STAR (v.2.7.6a), Samtools (v.1.7), CellPhoneDB (v. 3.1.0)<br>Code availability: The codes used for data analysis are available from the Zenodo repository (DOI:10.5281/zenodo.10651059).

For manuscripts utilizing custom algorithms or software that are central to the research but not yet described in published literature, software must be made available to editors and reviewers. We strongly encourage code deposition in a community repository (e.g. GitHub). See the Nature Portfolio guidelines for submitting code & software for further information.

Data

Policy information about availability of data

All manuscripts must include a data availability statement. This statement should provide the following information, where applicable:

- Accession codes, unique identifiers, or web links for publicly available datasets
- A description of any restrictions on data availability
- For clinical datasets or third party data, please ensure that the statement adheres to our policy

The scRNA-seq and spatial transcriptome datasets analyzed in this study are provided in Supplementary Data 1, 3, and 5, along with their accession codes and links. The processed scRNA-seq and spatial transcriptome data are available at Zenodo repository (DOI:10.5281/zenodo.10651059). Processed immunotherapy-treated lung cancer cohort data generated in this study have been deposited in the Gene Expression Omnibus repository under accession code GSE218989 [https://www.ncbi.nlm.nih.gov/geo/query/acc.cgi?acc=GSE218989]. Raw sequencing data of the immunotherapy-treated lung cancer cohort have been deposited in the European Genome-phenome Archive (EGA) under controlled access with accession number EGAD50000000469 [https://ega-archive.org/datasets/EGAD50000000469]. Data requests for academic or intellectual purposes will be reviewed by the data access committee, and are expected to be responded within 4 weeks. Our dataset can be interactively visualized at https://cellatlas.kaist.ac.kr/ecosystem/. Source data are provided with this paper.

Research involving human participants, their data, or biological material

Policy information about studies with human participants or human data. See also policy information about sex, gender (identity/presentation), and sexual orientation and race, ethnicity and racism.

Reporting on sex and gender	The findings of this study are expected to be generally applicable to both genders, as gender was not a consideration in the study design. Gender information was neither collected nor utilized in this study.
Reporting on race, ethnicity, or other socially relevant groupings	In this study, we did not use socially constructed or socially relevant categorizations based on race, ethnicity, or other social groupings for any analysis in this manuscript.
Population characteristics	A total of 497 immunotherapy-treated lung cancer patients samples were collected in this study. Histologically confirmed lung adenocarcinoma and lung squamous cell carcinoma patients, including previously reported cases, treated with either PD-1 or PD-L1 inhibitors were recruited.
Recruitment	A total of 497 histologically confirmed lung adenocarcinoma and lung squamous cell carcinoma patients treated with either PD-1 or PD-L1 inhibitors were recruited from Samsung Medical Center. Clinical information of this cohort was collected from electronic medical records and tumor response was evaluated with Response Evaluation Criteria in Solid Tumors (v1.1).
Ethics oversight	Our immunotherapy cohort study was approved by the Institutional Review Board of Samsung Medical Center (SMC 2018-03-130). All patients enrolled in the study provided informed written consent.

Note that full information on the approval of the study protocol must also be provided in the manuscript.

Field-specific reporting

Please select the one below that is the best fit for your research. If you are not sure, read the appropriate sections before making your selection.

☒ Life sciences ☐ Behavioural & social sciences ☐ Ecological, evolutionary & environmental sciences

For a reference copy of the document with all sections, see nature.com/documents/nr-reporting-summary-flat.pdf

Life sciences study design

All studies must disclose on these points even when the disclosure is negative.

Sample size	Sample size for scRNA-seq was determined by the publicly available resources.<br>No statistical tests were performed for sample size calculation
Data exclusions	All criteria for data exclusion were pre-established.<br>Studies that only include sorted cells (e.g.; CD45+ sorting), fluid samples (e.g.; ascites, CSF, or PBMC), cell-line cultures, mouse studies, and studies generated from nuclei-seq were excluded.
Replication	No replication was performed in this study.
Randomization	No randomization was performed in this study.
Blinding	No blinding was performed in this study.

Reporting for specific materials, systems and methods

We require information from authors about some types of materials, experimental systems and methods used in many studies. Here, indicate whether each material, system or method listed is relevant to your study. If you are not sure if a list item applies to your research, read the appropriate section before selecting a response.

Materials & experimental systems

n/a	Involved in the study
☒	☐ Antibodies
☒	☐ Eukaryotic cell lines
☒	☐ Palaeontology and archaeology
☒	☐ Animals and other organisms
☒	☐ Clinical data
☒	☐ Dual use research of concern
☒	☐ Plants

Methods

n/a	Involved in the study
☒	☐ ChIP-seq
☒	☐ Flow cytometry
☒	☐ MRI-based neuroimaging

Plants

Seed stocks	N/A
Novel plant genotypes	N/A
Authentication	N/A
