# Supplementary material for: Author Correction: Systematic dissection of tumor-normal single-cell ecosystems across a thousand tumors of 30 cancer types
Source: Nat Commun. 2025 Mar 21;16:2806. doi: 10.1038/s41467-025-58068-y (PMC11928607; doi:10.1038/s41467-025-58068-y)
Supplement: Supplementary file 1 — Original Figs. 5, 6, Supplementary Fig. 20 [file 41467_2025_58068_MOESM1_ESM.pdf]

Author Correction: Systematic dissection of tumor-normal single-cell ecosystems across a thousand tumors of 30 cancer types

Author Correction to: *Nature Communications* https://doi.org/10.1038/s41467-024-48310-4, published online 14 May 2024

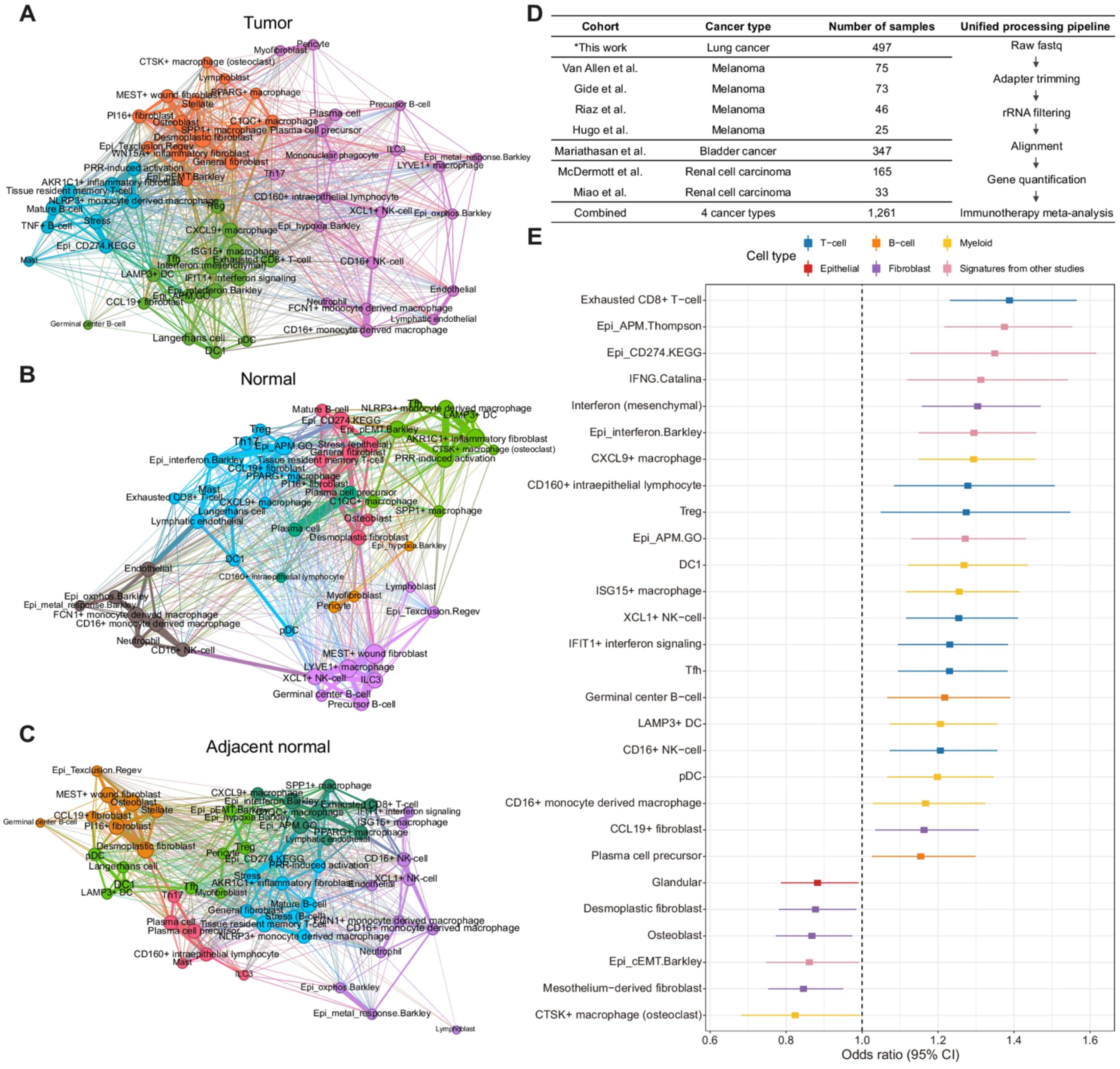

Original Fig. 5

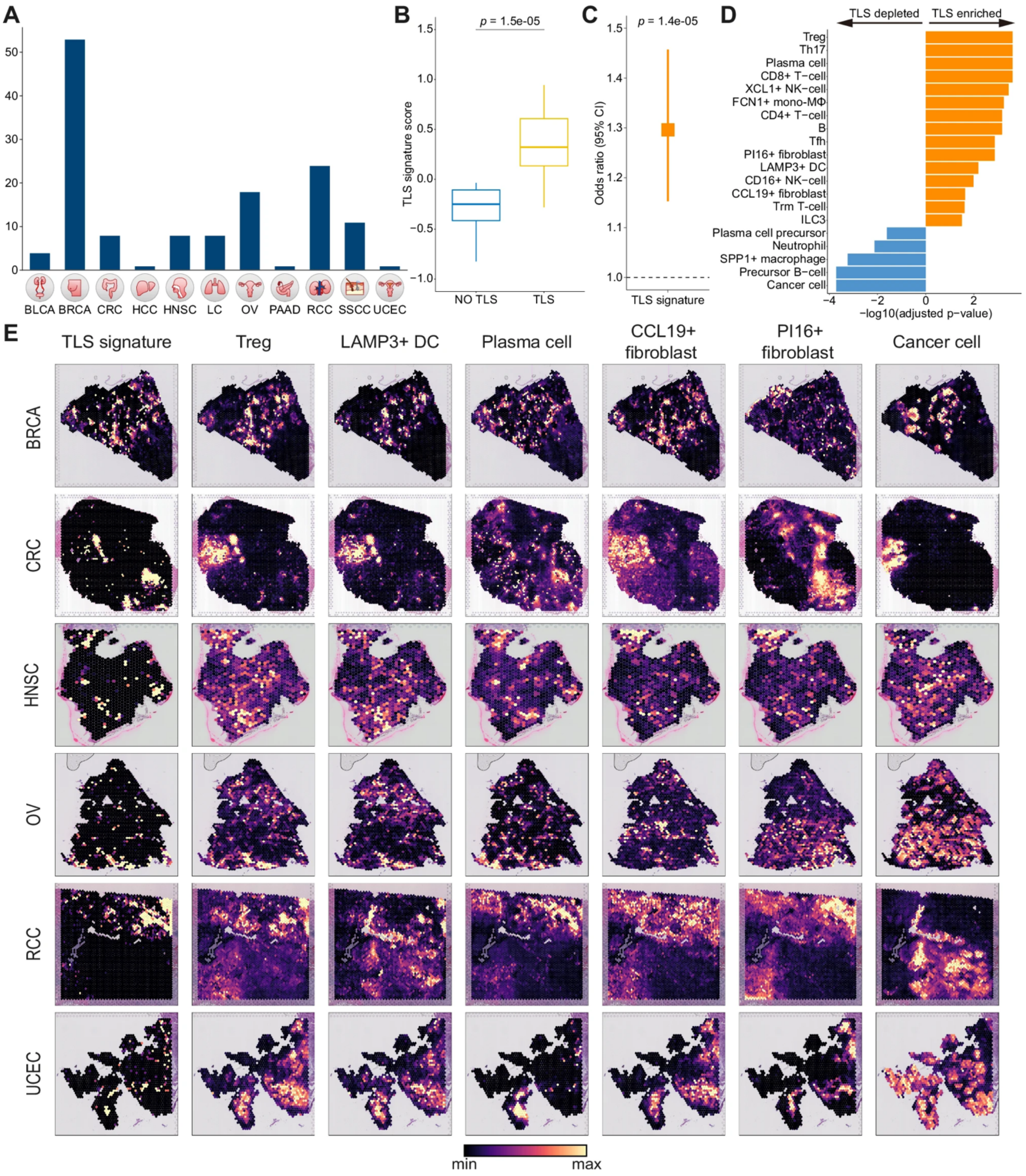

Original Fig. 6

Figure S20

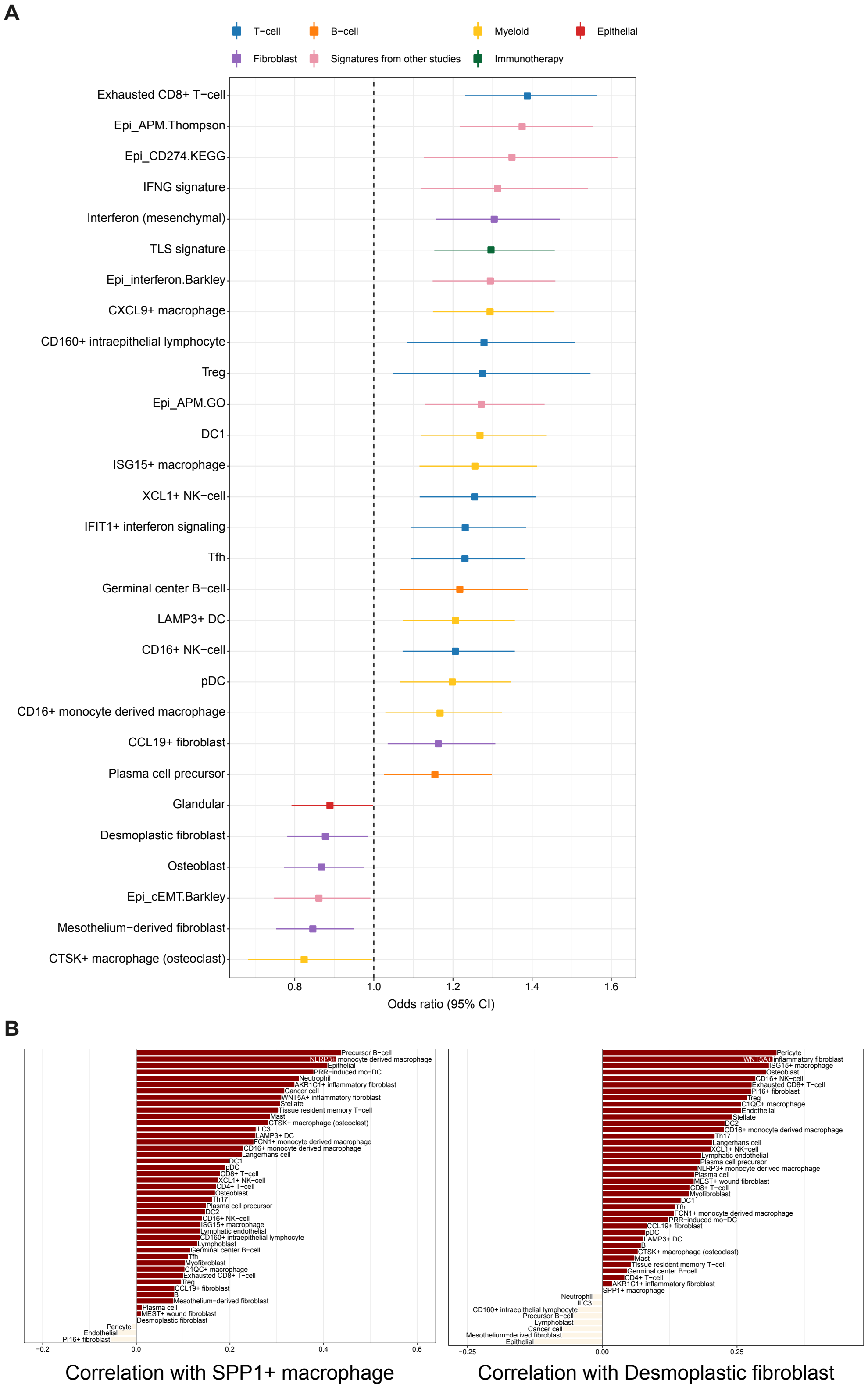

Original Supplementary Fig. 20
