# Supplementary material for: Author Correction: Systematic dissection of tumor-normal single-cell ecosystems across a thousand tumors of 30 cancer types
Source: Nat Commun. 2025 Mar 21;16:2806. doi: 10.1038/s41467-025-58068-y (PMC11928607; doi:10.1038/s41467-025-58068-y)

Figure S1

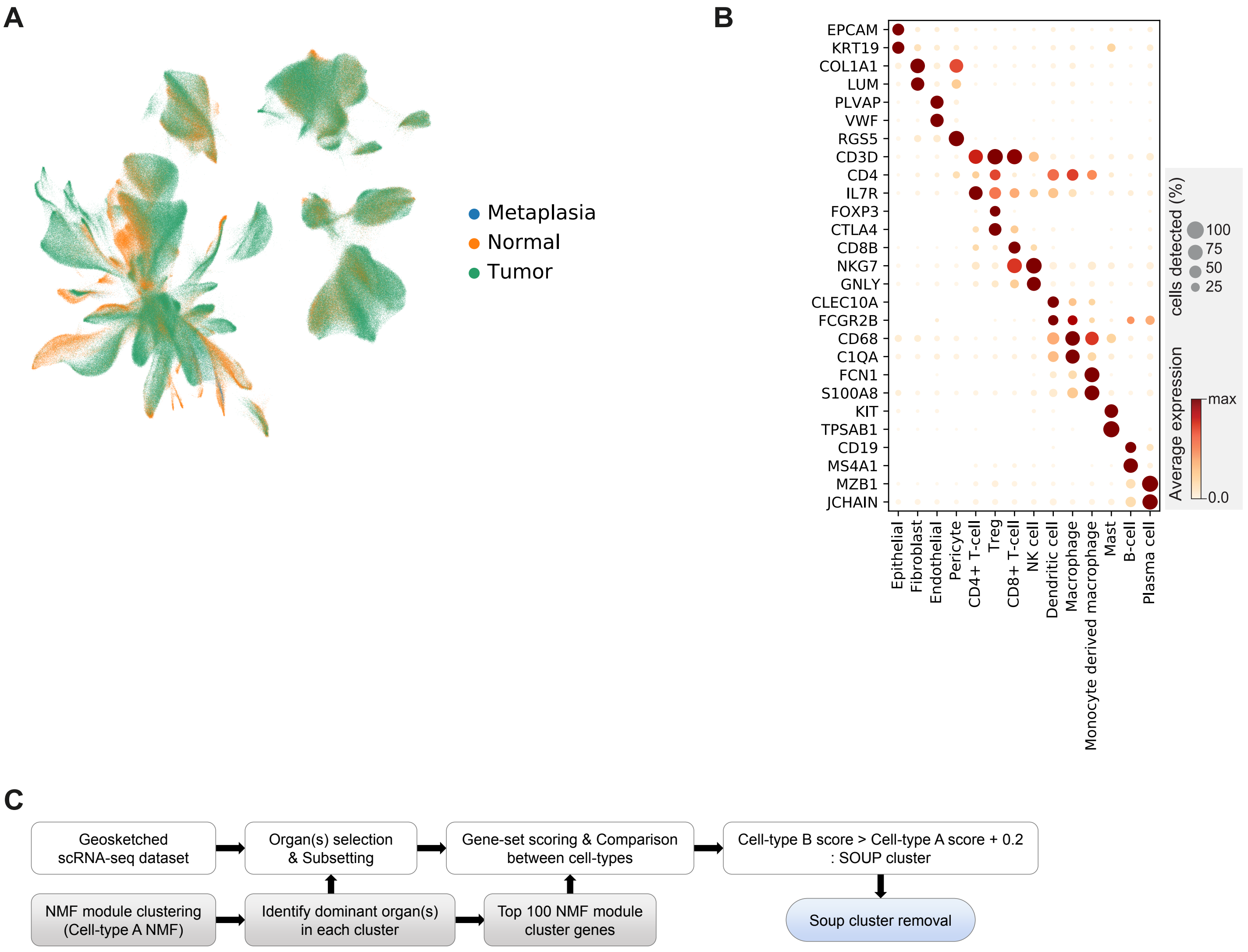

A
Metaplasia
Normal
Tumor
B
EPCAM
KRT19
COL1A1
LUM
PLVAP
VWF
RGS5
CD3D
CD4
IL7R
FOXP3
CTLA4
CD8B
NKG7
GNLY
CLEC10A
FCGR2B
CD68
C1QA
FCN1
S100A8
KIT
TPSAB1
CD19
MS4A1
MZB1
JCHAIN
Epithelial
Fibroblast
Endothelial
Pericyte
CD4+ T-cell
Treg
CD8+ T-cell
NK cell
Dendritic cell
Macrophage
Monocyte derived macrophage
Mast
B-cell
Plasma cell
cells detected (%)
100
75
50
25
Average expression
max
0.0
C
Geosketched scRNA-seq dataset
Organ(s) selection & Subsetting
Gene-set scoring & Comparison between cell-types
Cell-type B score > Cell-type A score + 0.2 : SOUP cluster
NMF module clustering (Cell-type A NMF)
Identify dominant organ(s) in each cluster
Top 100 NMF module cluster genes
Soup cluster removal

Figure S2

A

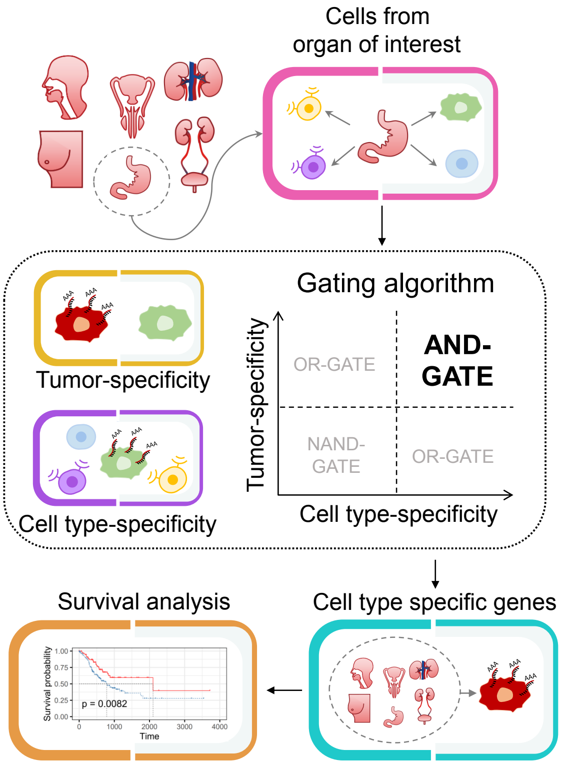

Cells from organ of interest
Gating algorithm
Tumor-specificity
Cell type-specificity
OR-GATE
AND-GATE
NAND-GATE
OR-GATE
Tumor-specificity
Cell type-specificity
Survival analysis
Cell type specific genes
p = 0.0082
Survival probability
Time

B

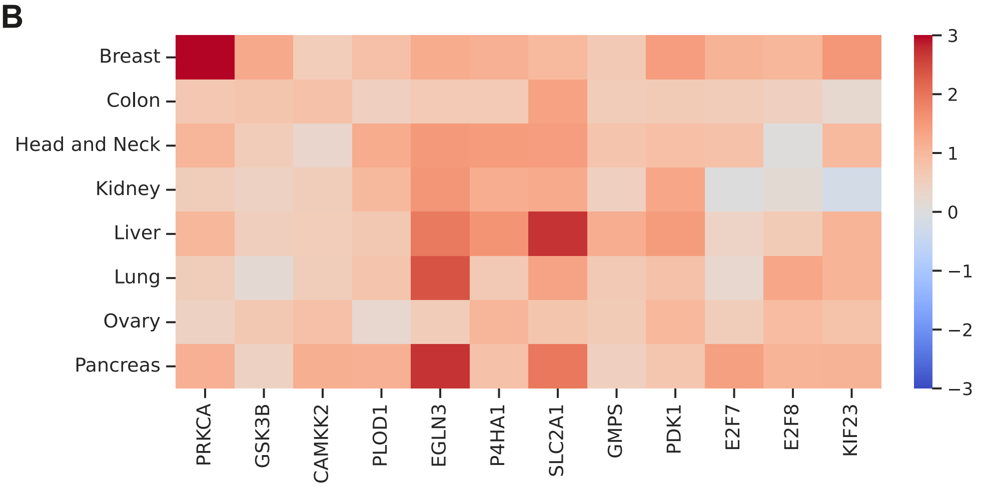

Breast
Colon
Head and Neck
Kidney
Liver
Lung
Ovary
Pancreas
PRKCA
GSK3B
CAMKK2
PLOD1
EGLN3
P4HA1
SLC2A1
GMPS
PDK1
E2F7
E2F8
KIF23
3
2
1
0
−1
−2
−3

Figure S3

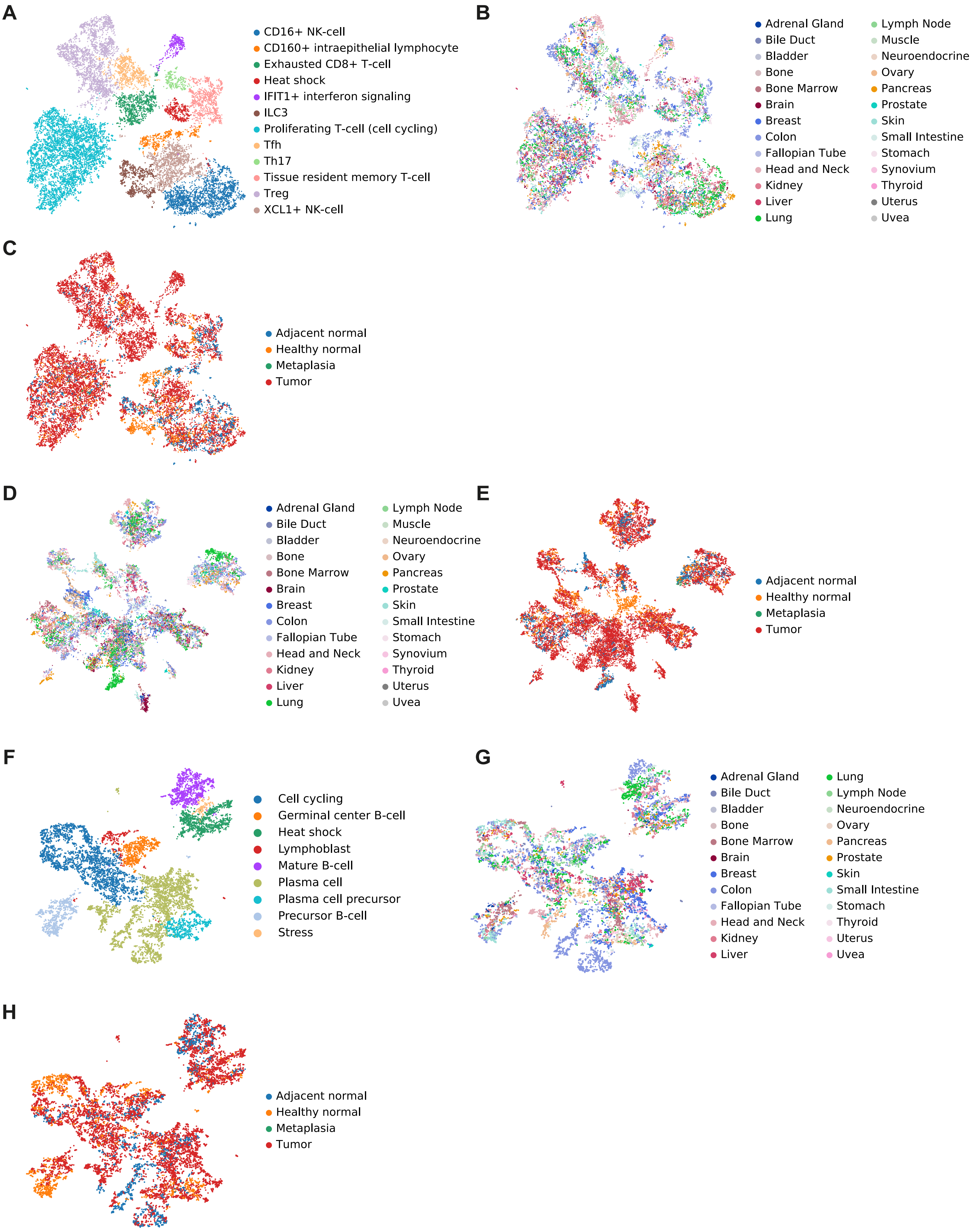
A
CD16+ NK-cell
CD160+ intraepithelial lymphocyte
Exhausted CD8+ T-cell
Heat shock
IFIT1+ interferon signaling
ILC3
Proliferating T-cell (cell cycling)
Tfh
Th17
Tissue resident memory T-cell
Treg
XCL1+ NK-cell
B
Adrenal Gland
Bile Duct
Bladder
Bone
Bone Marrow
Brain
Breast
Colon
Fallopian Tube
Head and Neck
Kidney
Liver
Lung
Lymph Node
Muscle
Neuroendocrine
Ovary
Pancreas
Prostate
Skin
Small Intestine
Stomach
Synovium
Thyroid
Uterus
Uvea
C
Adjacent normal
Healthy normal
Metaplasia
Tumor
D
Adrenal Gland
Bile Duct
Bladder
Bone
Bone Marrow
Brain
Breast
Colon
Fallopian Tube
Head and Neck
Kidney
Liver
Lung
Lymph Node
Muscle
Neuroendocrine
Ovary
Pancreas
Prostate
Skin
Small Intestine
Stomach
Synovium
Thyroid
Uterus
Uvea
E
Adjacent normal
Healthy normal
Metaplasia
Tumor
F
Cell cycling
Germinal center B-cell
Heat shock
Lymphoblast
Mature B-cell
Plasma cell
Plasma cell precursor
Precursor B-cell
Stress
G
Adrenal Gland
Bile Duct
Bladder
Bone
Bone Marrow
Brain
Breast
Colon
Fallopian Tube
Head and Neck
Kidney
Liver
Lung
Lymph Node
Neuroendocrine
Ovary
Pancreas
Prostate
Skin
Small Intestine
Stomach
Thyroid
Uterus
Uvea
H
Adjacent normal
Healthy normal
Metaplasia
Tumor

Figure S4

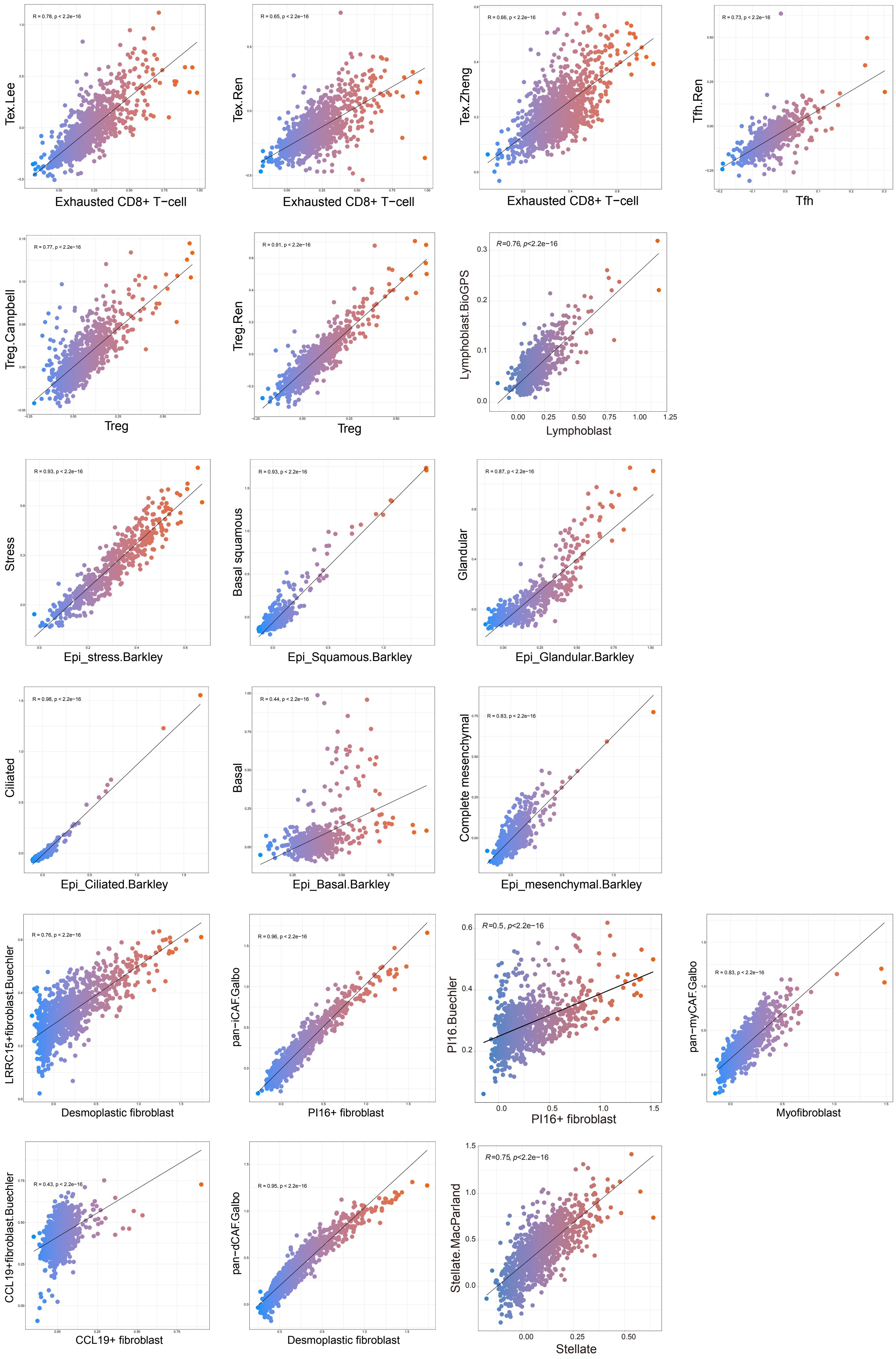
R = 0.78, p < 2.2e−16
Tex.Lee
Exhausted CD8+ T−cell
R = 0.65, p < 2.2e−16
Tex.Ren
Exhausted CD8+ T−cell
R = 0.66, p < 2.2e−16
Tex.Zheng
Exhausted CD8+ T−cell
R = 0.73, p < 2.2e−16
Tfh.Ren
Tfh
R = 0.77, p < 2.2e−16
Treg.Campbell
Treg
R = 0.91, p < 2.2e−16
Treg.Ren
Treg
R=0.76, p<2.2e−16
Lymphoblast.BioGPS
Lymphoblast
R = 0.93, p < 2.2e−16
Stress
Epi_stress.Barkley
R = 0.93, p < 2.2e−16
Basal squamous
Epi_Squamous.Barkley
R = 0.87, p < 2.2e−16
Glandular
Epi_Glandular.Barkley
R = 0.98, p < 2.2e−16
Ciliated
Epi_Ciliated.Barkley
R = 0.44, p < 2.2e−16
Basal
Epi_Basal.Barkley
R = 0.83, p < 2.2e−16
Complete mesenchymal
Epi_mesenchymal.Barkley
R = 0.76, p < 2.2e−16
LRRC15+fibroblast.Buechler
Desmoplastic fibroblast
R = 0.96, p < 2.2e−16
pan−iCAF.Galbo
PI16+ fibroblast
R=0.5, p<2.2e−16
PI16.Buechler
PI16+ fibroblast
R = 0.83, p < 2.2e−16
pan−myCAF.Galbo
Myofibroblast
R = 0.43, p < 2.2e−16
CCL19+fibroblast.Buechler
CCL19+ fibroblast
R = 0.95, p < 2.2e−16
pan−dCAF.Galbo
Desmoplastic fibroblast
R=0.75, p<2.2e−16
Stellate.MacParland
Stellate

Figure S5

A

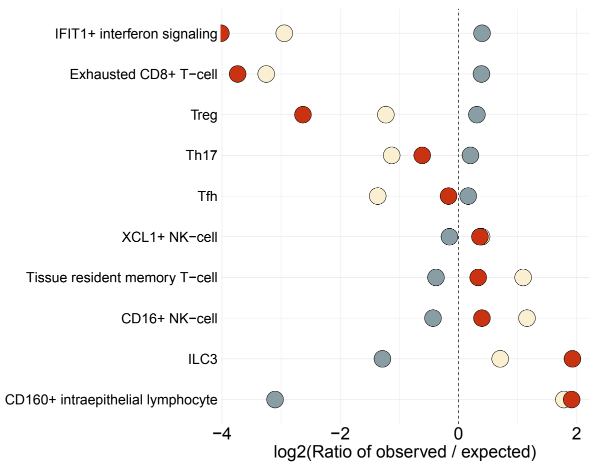

B

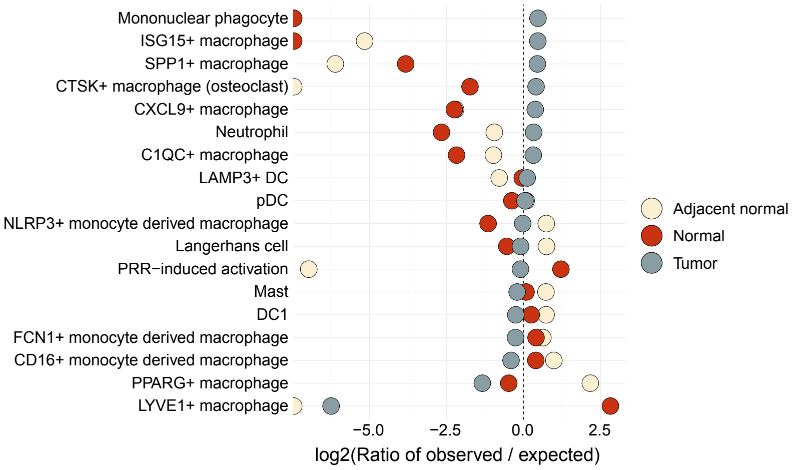

C

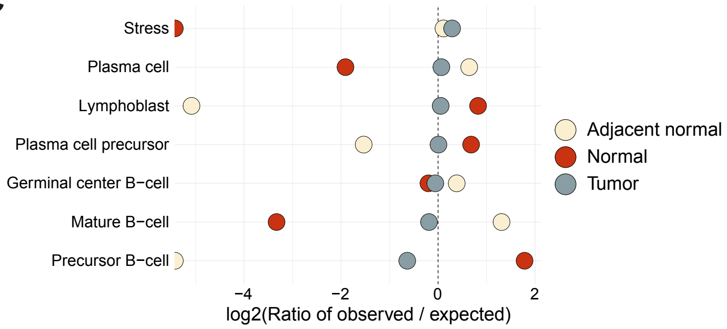

Figure S6

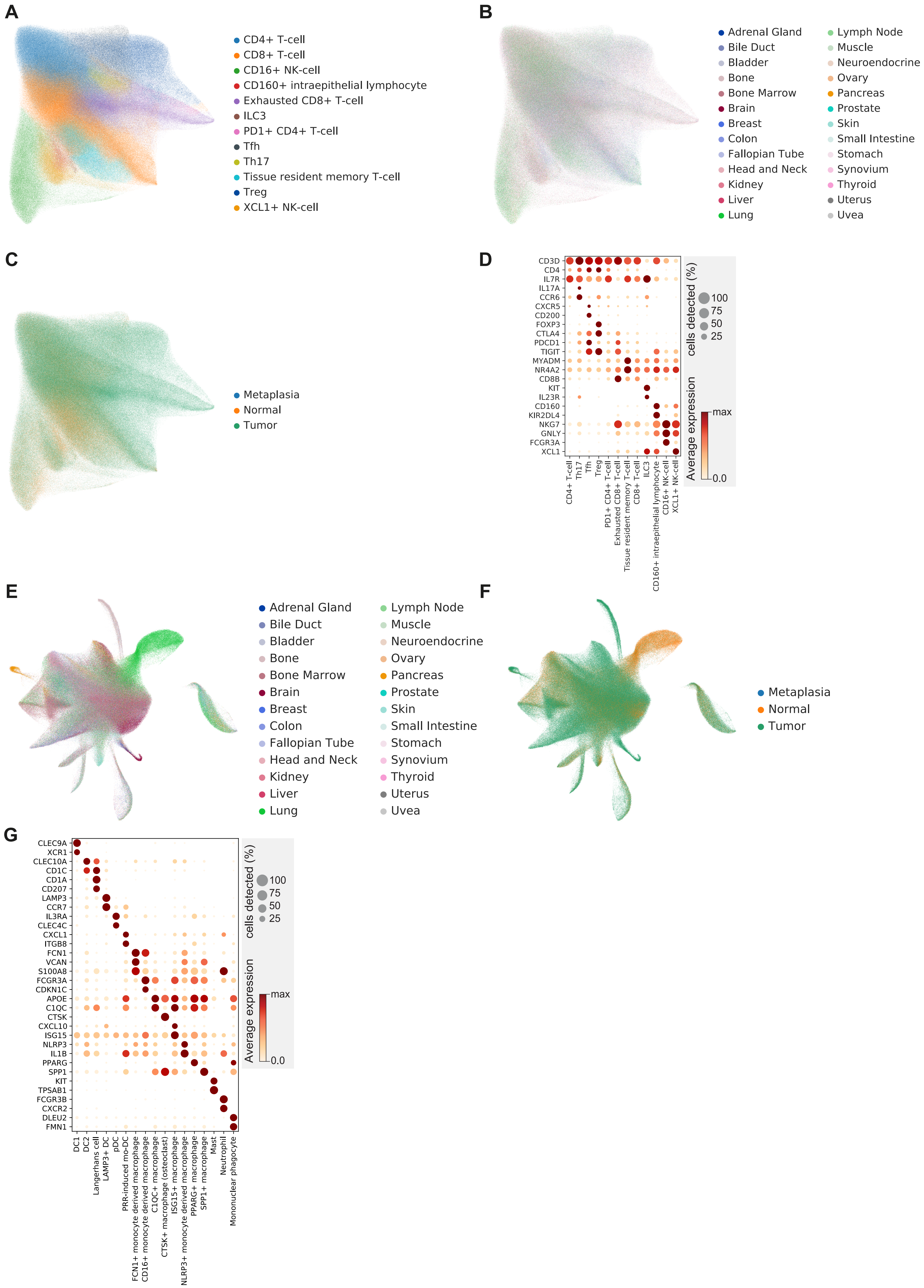

A
CD4+ T-cell
CD8+ T-cell
CD16+ NK-cell
CD160+ intraepithelial lymphocyte
Exhausted CD8+ T-cell
ILC3
PD1+ CD4+ T-cell
Tfh
Th17
Tissue resident memory T-cell
Treg
XCL1+ NK-cell
B
Adrenal Gland
Bile Duct
Bladder
Bone
Bone Marrow
Brain
Breast
Colon
Fallopian Tube
Head and Neck
Kidney
Liver
Lung
Lymph Node
Muscle
Neuroendocrine
Ovary
Pancreas
Prostate
Skin
Small Intestine
Stomach
Synovium
Thyroid
Uterus
Uvea
C
Metaplasia
Normal
Tumor
D
CD3D
CD4
IL7R
IL17A
CCR6
CXCR5
CD200
FOXP3
CTLA4
PDCD1
TIGIT
MYADM
NR4A2
CD8B
KIT
IL23R
CD160
KIR2DL4
NKG7
GNLY
FCGR3A
XCL1
CD4+ T-cell
Th17
Tfh
Treg
PD1+ CD4+ T-cell
Exhausted CD8+ T-cell
Tissue resident memory T-cell
CD8+ T-cell
ILC3
CD160+ intraepithelial lymphocyte
CD16+ NK-cell
XCL1+ NK-cell
cells detected (%)
100
75
50
25
Average expression
max
0.0
E
Adrenal Gland
Bile Duct
Bladder
Bone
Bone Marrow
Brain
Breast
Colon
Fallopian Tube
Head and Neck
Kidney
Liver
Lung
Lymph Node
Muscle
Neuroendocrine
Ovary
Pancreas
Prostate
Skin
Small Intestine
Stomach
Synovium
Thyroid
Uterus
Uvea
F
Metaplasia
Normal
Tumor
G
CLEC9A
XCR1
CLEC10A
CD1C
CD1A
CD207
LAMP3
CCR7
IL3RA
CLEC4C
CXCL1
ITGB8
FCN1
VCAN
S100A8
FCGR3A
CDKN1C
APOE
C1QC
CTSK
CXCL10
ISG15
NLRP3
IL1B
PPARG
SPP1
KIT
TPSAB1
FCGR3B
CXCR2
DLEU2
FMN1
DC1
DC2
Langerhans cell
LAMP3+ DC
pDC
PRR-induced mo-DC
FCN1+ monocyte derived macrophage
CD16+ monocyte derived macrophage
C1QC+ macrophage
CTSK+ macrophage (osteoclast)
ISG15+ macrophage
NLRP3+ monocyte derived macrophage
PPARG+ macrophage
SPP1+ macrophage
Mast
Neutrophil
Mononuclear phagocyte
cells detected (%)
100
75
50
25
Average expression
max
0.0

Figure S7

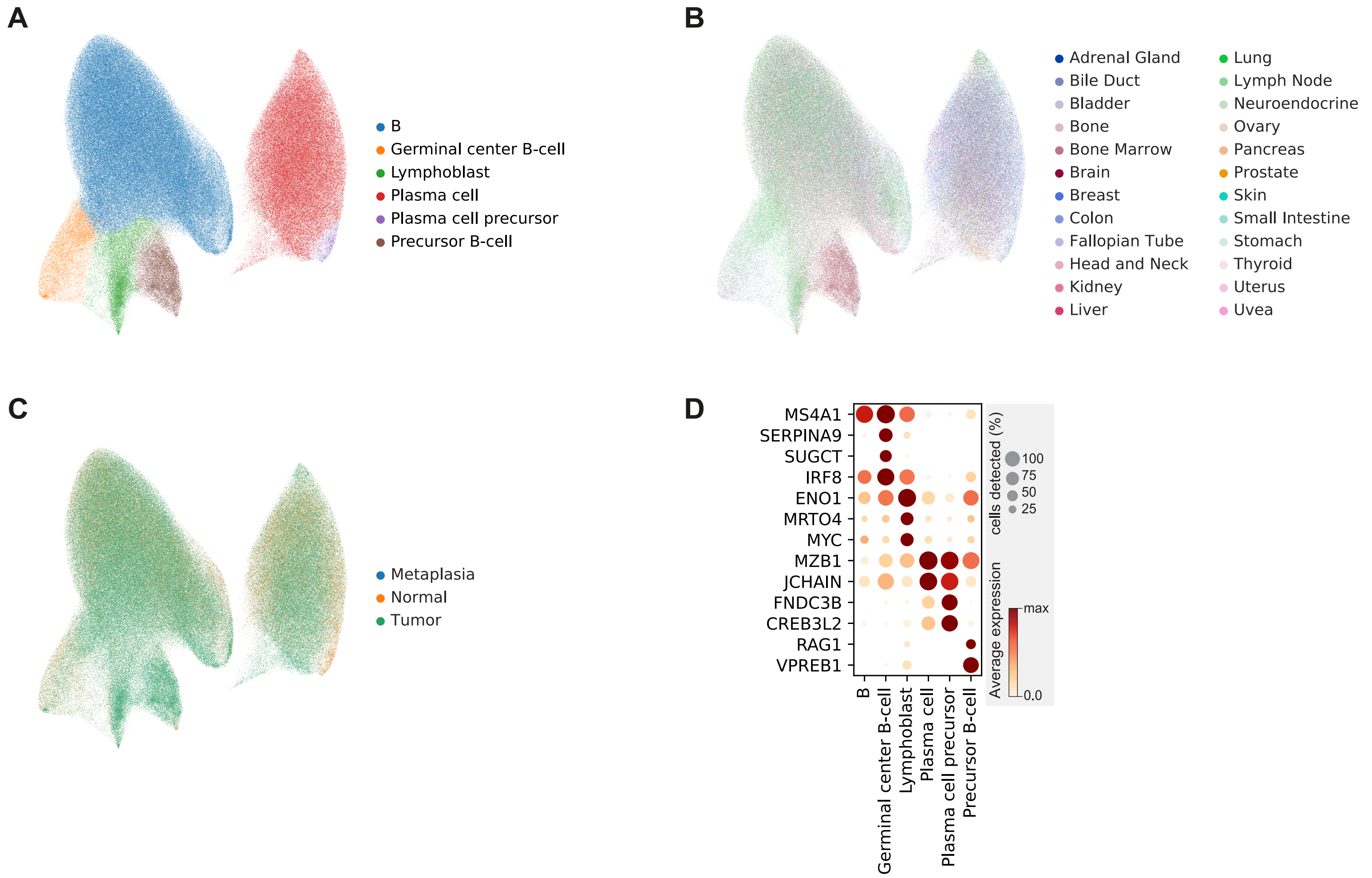

A
B
Germinal center B-cell
Lymphoblast
Plasma cell
Plasma cell precursor
Precursor B-cell
B
Adrenal Gland
Bile Duct
Bladder
Bone
Bone Marrow
Brain
Breast
Colon
Fallopian Tube
Head and Neck
Kidney
Liver
Lung
Lymph Node
Neuroendocrine
Ovary
Pancreas
Prostate
Skin
Small Intestine
Stomach
Thyroid
Uterus
Uvea
C
Metaplasia
Normal
Tumor
D
MS4A1
SERPINA9
SUGCT
IRF8
ENO1
MRTO4
MYC
MZB1
JCHAIN
FNDC3B
CREB3L2
RAG1
VPREB1
B
Germinal center B-cell
Lymphoblast
Plasma cell
Plasma cell precursor
Precursor B-cell
cells detected (%)
100
75
50
25
Average expression
max
0.0

Figure S8

A

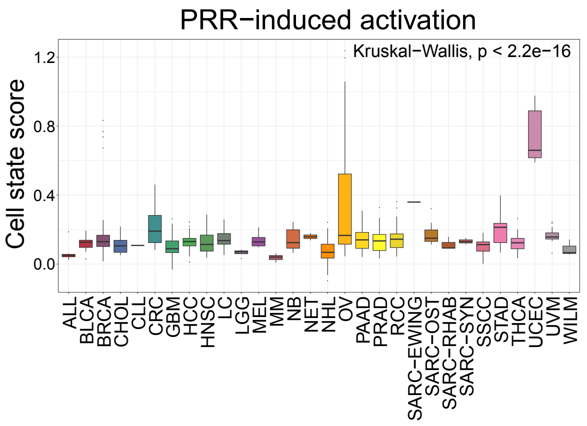

PRR−induced activation
Kruskal−Wallis, p < 2.2e−16
Cell state score
1.2
0.8
0.4
0.0
ALL
BLCA
BRCA
CHOL
CLL
CRC
GBM
HCC
HNSC
LC
LGG
MEL
MM
NB
NET
NHL
OV
PAAD
PRAD
RCC
SARC−EWING
SARC−OST
SARC−RHAB
SARC−SYN
SSCC
STAD
THCA
UCEC
UVM
WILM

B

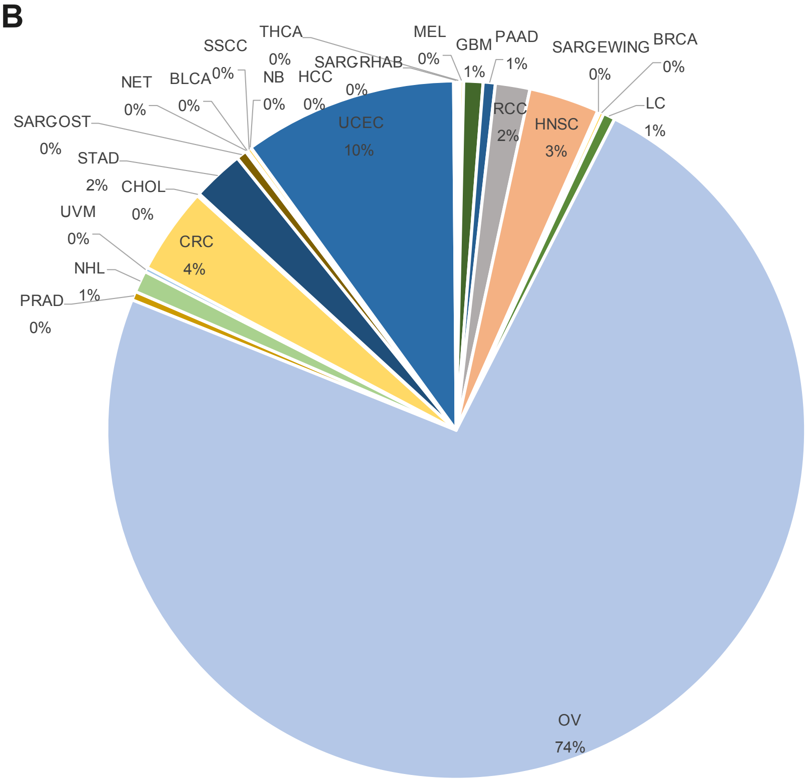

THCA 0%
SSCC 0%
MEL 0%
GBM 1%
PAAD 1%
SARGEWING 0%
BRCA 0%
SARGRHAB 0%
NET 0%
BLCA 0%
NB 0%
HCC 0%
UCEC 10%
RCC 2%
HNSC 3%
LC 1%
SARGOST 0%
STAD 2%
CHOL 0%
UVM 0%
CRC 4%
NHL 1%
PRAD 0%
OV 74%

Figure S9

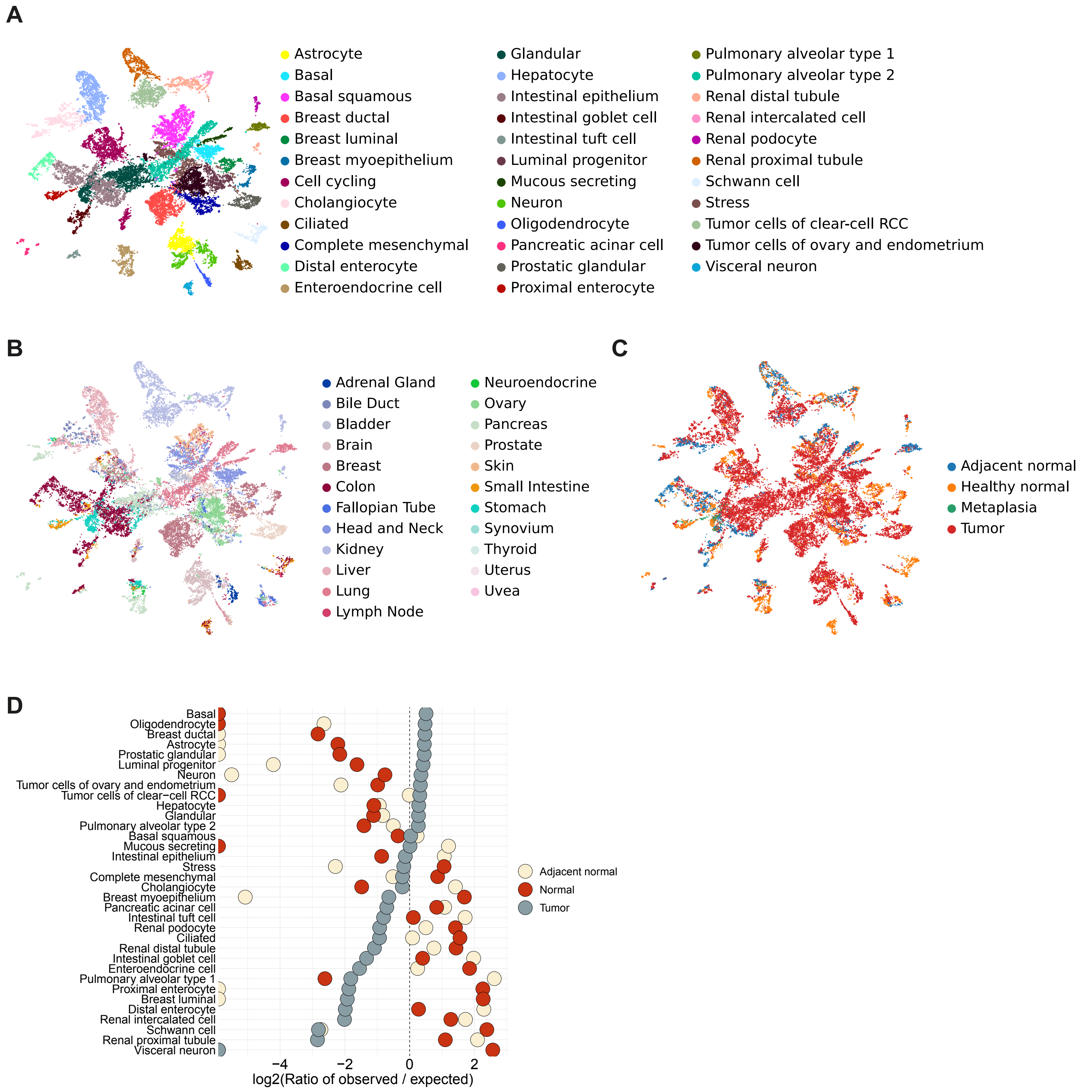
A
Astrocyte
Basal
Basal squamous
Breast ductal
Breast luminal
Breast myoepithelium
Cell cycling
Cholangiocyte
Ciliated
Complete mesenchymal
Distal enterocyte
Enteroendocrine cell
Glandular
Hepatocyte
Intestinal epithelium
Intestinal goblet cell
Intestinal tuft cell
Luminal progenitor
Mucous secreting
Neuron
Oligodendrocyte
Pancreatic acinar cell
Prostatic glandular
Proximal enterocyte
Pulmonary alveolar type 1
Pulmonary alveolar type 2
Renal distal tubule
Renal intercalated cell
Renal podocyte
Renal proximal tubule
Schwann cell
Stress
Tumor cells of clear-cell RCC
Tumor cells of ovary and endometrium
Visceral neuron
B
Adrenal Gland
Bile Duct
Bladder
Brain
Breast
Colon
Fallopian Tube
Head and Neck
Kidney
Liver
Lung
Lymph Node
Neuroendocrine
Ovary
Pancreas
Prostate
Skin
Small Intestine
Stomach
Synovium
Thyroid
Uterus
Uvea
C
Adjacent normal
Healthy normal
Metaplasia
Tumor
D
Basal
Oligodendrocyte
Breast ductal
Astrocyte
Prostatic glandular
Luminal progenitor
Neuron
Tumor cells of ovary and endometrium
Tumor cells of clear−cell RCC
Hepatocyte
Glandular
Pulmonary alveolar type 2
Basal squamous
Mucous secreting
Intestinal epithelium
Stress
Complete mesenchymal
Cholangiocyte
Breast myoepithelium
Pancreatic acinar cell
Intestinal tuft cell
Renal podocyte
Ciliated
Renal distal tubule
Intestinal goblet cell
Enteroendocrine cell
Pulmonary alveolar type 1
Proximal enterocyte
Breast luminal
Distal enterocyte
Renal intercalated cell
Schwann cell
Renal proximal tubule
Visceral neuron
−4
−2
0
2
log2(Ratio of observed / expected)
Adjacent normal
Normal
Tumor

Figure S10

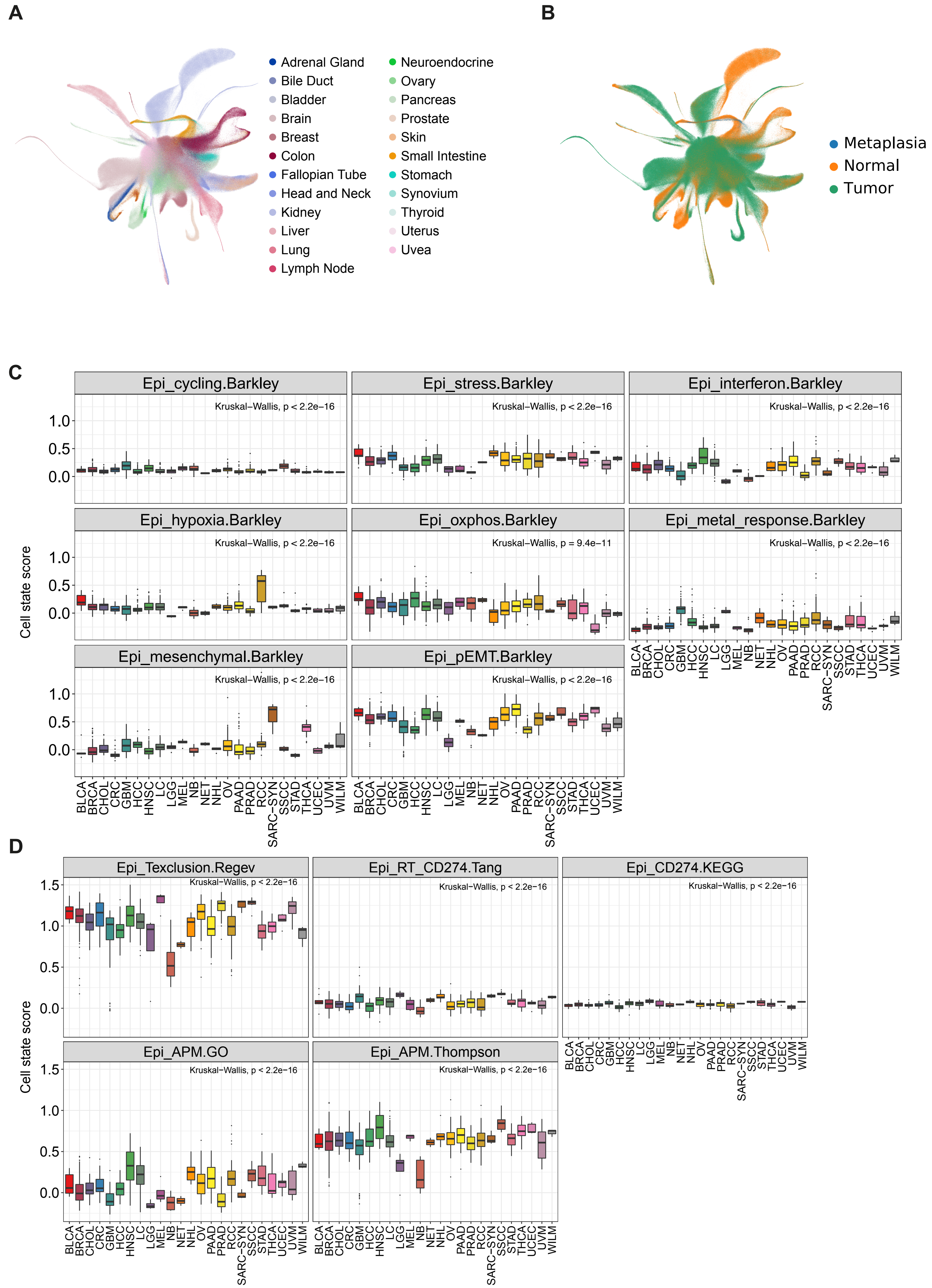
A
B
Adrenal Gland
Bile Duct
Bladder
Brain
Breast
Colon
Fallopian Tube
Head and Neck
Kidney
Liver
Lung
Lymph Node
Neuroendocrine
Ovary
Pancreas
Prostate
Skin
Small Intestine
Stomach
Synovium
Thyroid
Uterus
Uvea
Metaplasia
Normal
Tumor
C
Epi_cycling.Barkley
Kruskal–Wallis, p < 2.2e−16
Epi_stress.Barkley
Kruskal–Wallis, p < 2.2e−16
Epi_interferon.Barkley
Kruskal–Wallis, p < 2.2e−16
Epi_hypoxia.Barkley
Kruskal–Wallis, p < 2.2e−16
Epi_oxphos.Barkley
Kruskal–Wallis, p = 9.4e−11
Epi_metal_response.Barkley
Kruskal–Wallis, p < 2.2e−16
Epi_mesenchymal.Barkley
Kruskal–Wallis, p < 2.2e−16
Epi_pEMT.Barkley
Kruskal–Wallis, p < 2.2e−16
Cell state score
0.0
0.5
1.0
BLCA
BRCA
CHOL
CRC
GBM
HCC
HNSC
LC
LGG
MEL
NB
NET
NHL
OV
PAAD
PRAD
RCC
SARC−SYN
SSCC
STAD
THCA
UCEC
UVM
WILM
D
Epi_Texclusion.Regev
Kruskal–Wallis, p < 2.2e−16
Epi_RT_CD274.Tang
Kruskal–Wallis, p < 2.2e−16
Epi_CD274.KEGG
Kruskal–Wallis, p < 2.2e−16
Epi_APM.GO
Kruskal–Wallis, p < 2.2e−16
Epi_APM.Thompson
Kruskal–Wallis, p < 2.2e−16
Cell state score
0.0
0.5
1.0
1.5

Figure S11

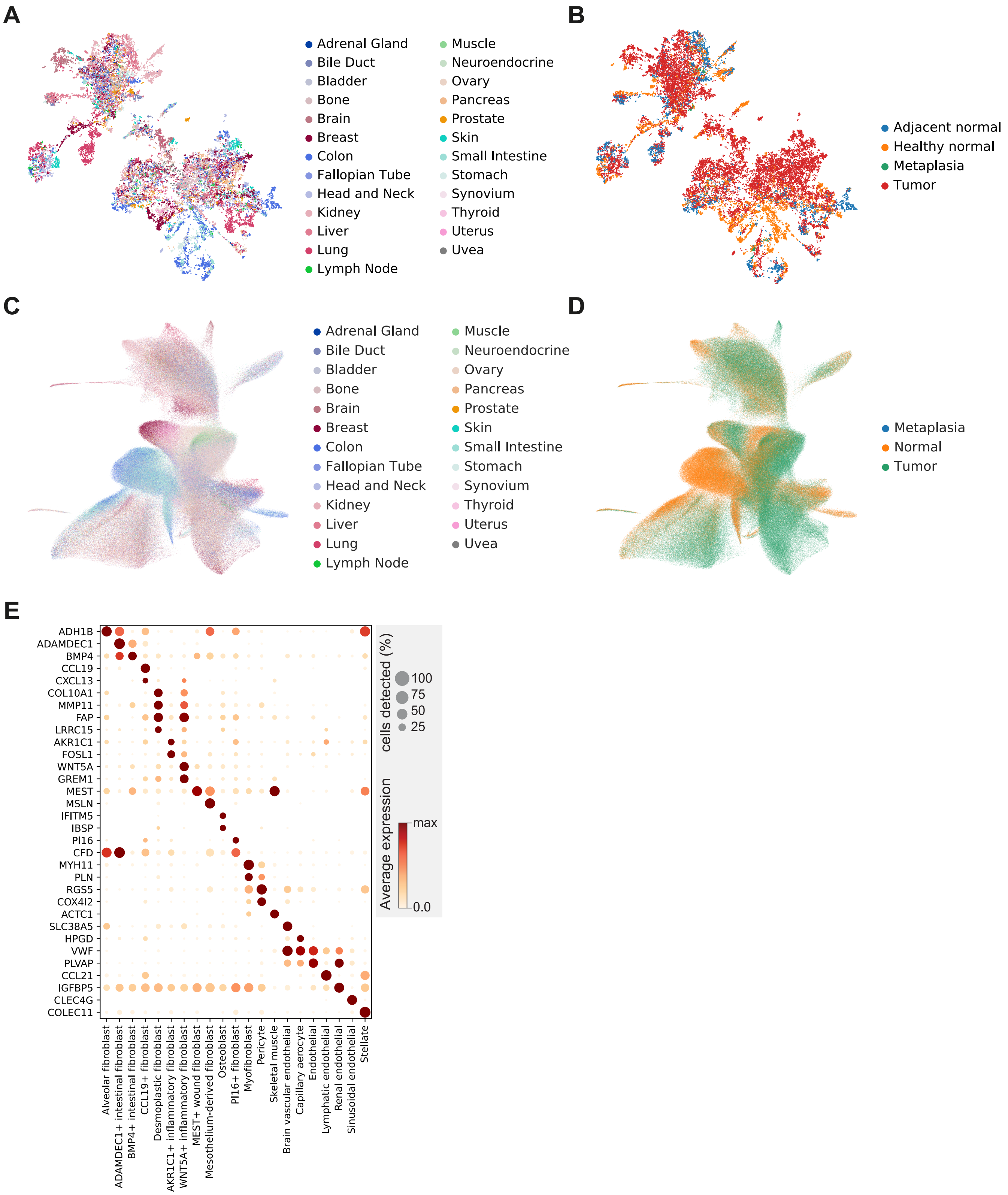
A
Adrenal Gland
Bile Duct
Bladder
Bone
Brain
Breast
Colon
Fallopian Tube
Head and Neck
Kidney
Liver
Lung
Lymph Node
Muscle
Neuroendocrine
Ovary
Pancreas
Prostate
Skin
Small Intestine
Stomach
Synovium
Thyroid
Uterus
Uvea
B
Adjacent normal
Healthy normal
Metaplasia
Tumor
C
Adrenal Gland
Bile Duct
Bladder
Bone
Brain
Breast
Colon
Fallopian Tube
Head and Neck
Kidney
Liver
Lung
Lymph Node
Muscle
Neuroendocrine
Ovary
Pancreas
Prostate
Skin
Small Intestine
Stomach
Synovium
Thyroid
Uterus
Uvea
D
Metaplasia
Normal
Tumor
E
ADH1B
ADAMDEC1
BMP4
CCL19
CXCL13
COL10A1
MMP11
FAP
LRRC15
AKR1C1
FOSL1
WNT5A
GREM1
MEST
MSLN
IFITM5
IBSP
PI16
CFD
MYH11
PLN
RGS5
COX4I2
ACTC1
SLC38A5
HPGD
VWF
PLVAP
CCL21
IGFBP5
CLEC4G
COLEC11
Alveolar fibroblast
ADAMDEC1+ intestinal fibroblast
BMP4+ intestinal fibroblast
CCL19+ fibroblast
Desmoplastic fibroblast
AKR1C1+ inflammatory fibroblast
WNT5A+ inflammatory fibroblast
MEST+ wound fibroblast
Mesothelium-derived fibroblast
Osteoblast
PI16+ fibroblast
Myofibroblast
Pericyte
Skeletal muscle
Brain vascular endothelial
Capillary aerocyte
Endothelial
Lymphatic endothelial
Renal endothelial
Sinusoidal endothelial
Stellate
cells detected (%)
100
75
50
25
Average expression
max
0.0

Figure S12

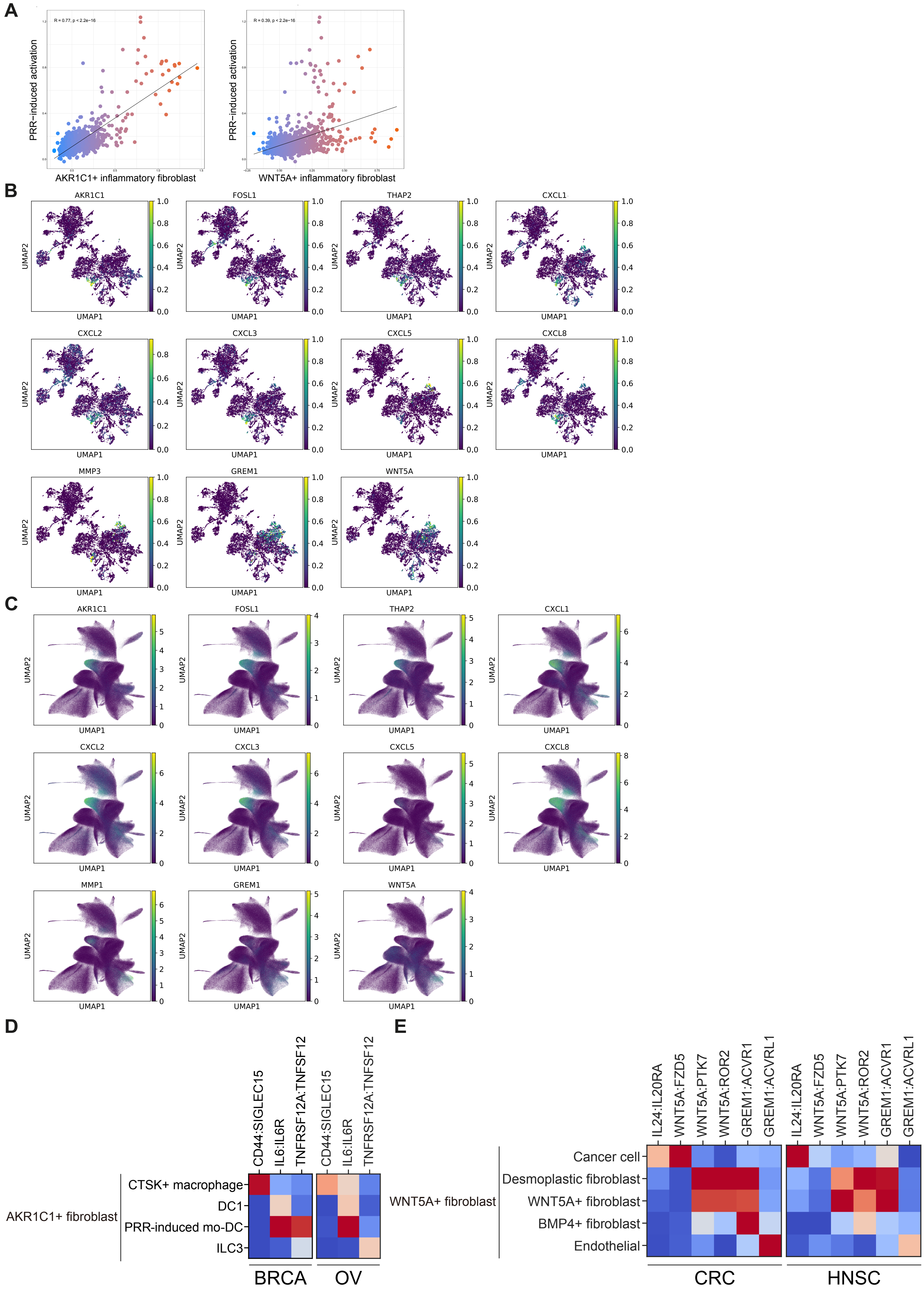
A
R = 0.77, p < 2.2e−16
PRR−induced activation
AKR1C1+ inflammatory fibroblast
R = 0.39, p < 2.2e−16
PRR−induced activation
WNT5A+ inflammatory fibroblast
B
AKR1C1
FOSL1
THAP2
CXCL1
CXCL2
CXCL3
CXCL5
CXCL8
MMP3
GREM1
WNT5A
UMAP1
UMAP2
C
AKR1C1
FOSL1
THAP2
CXCL1
CXCL2
CXCL3
CXCL5
CXCL8
MMP1
GREM1
WNT5A
UMAP1
UMAP2
D
CD44:SIGLEC15
IL6:IL6R
TNFRSF12A:TNFSF12
AKR1C1+ fibroblast
CTSK+ macrophage
DC1
PRR-induced mo-DC
ILC3
BRCA
OV
E
IL24:IL20RA
WNT5A:FZD5
WNT5A:PTK7
WNT5A:ROR2
GREM1:ACVR1
GREM1:ACVRL1
WNT5A+ fibroblast
Cancer cell
Desmoplastic fibroblast
WNT5A+ fibroblast
BMP4+ fibroblast
Endothelial
CRC
HNSC

Figure S13

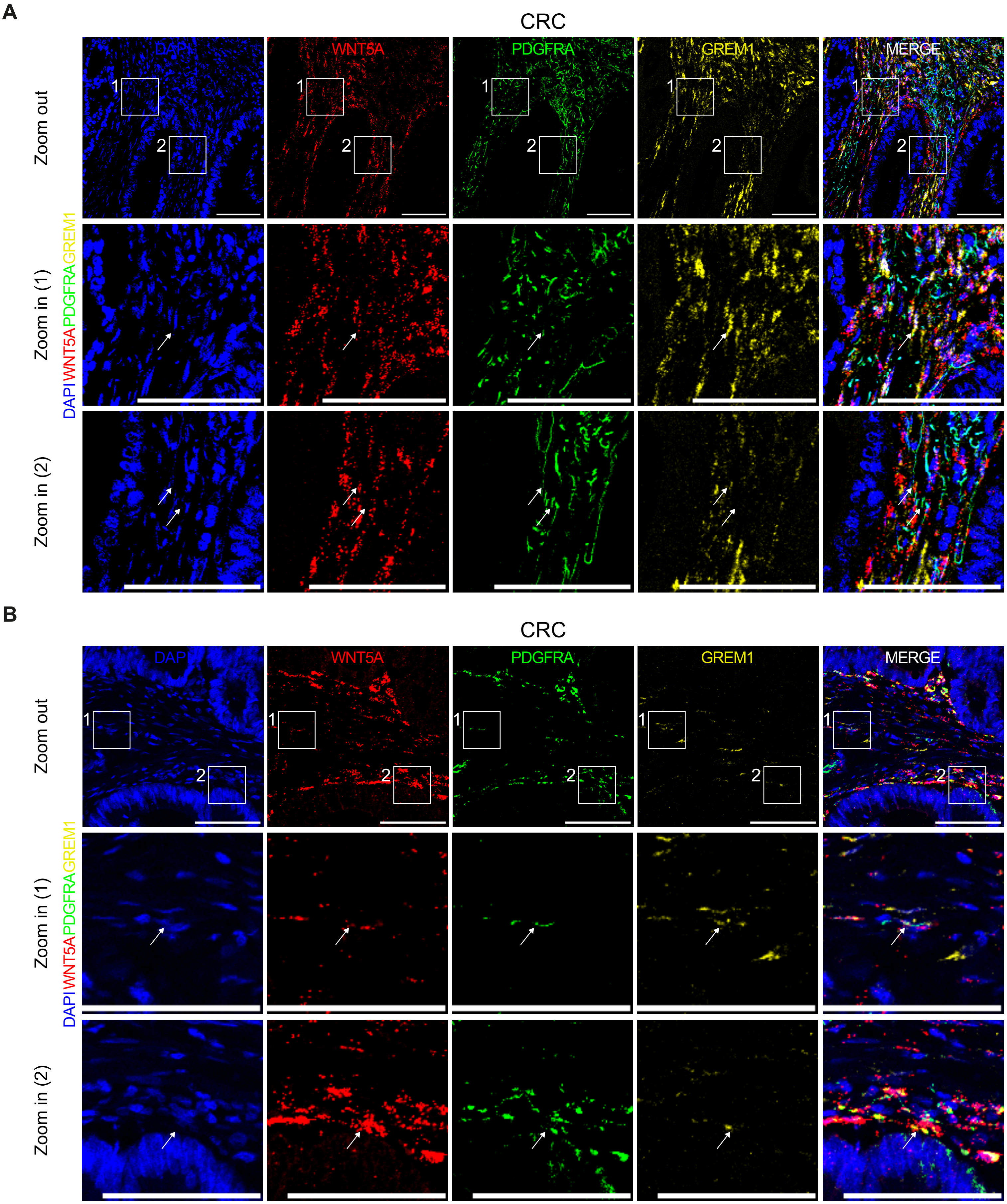
A
CRC
DAPI
WNT5A
PDGFRA
GREM1
MERGE
Zoom out
Zoom in (1)
Zoom in (2)
DAPI WNT5A PDGFRA GREM1
1
2
B
CRC
DAPI
WNT5A
PDGFRA
GREM1
MERGE
Zoom out
Zoom in (1)
Zoom in (2)
DAPI WNT5A PDGFRA GREM1
1
2

Figure S14

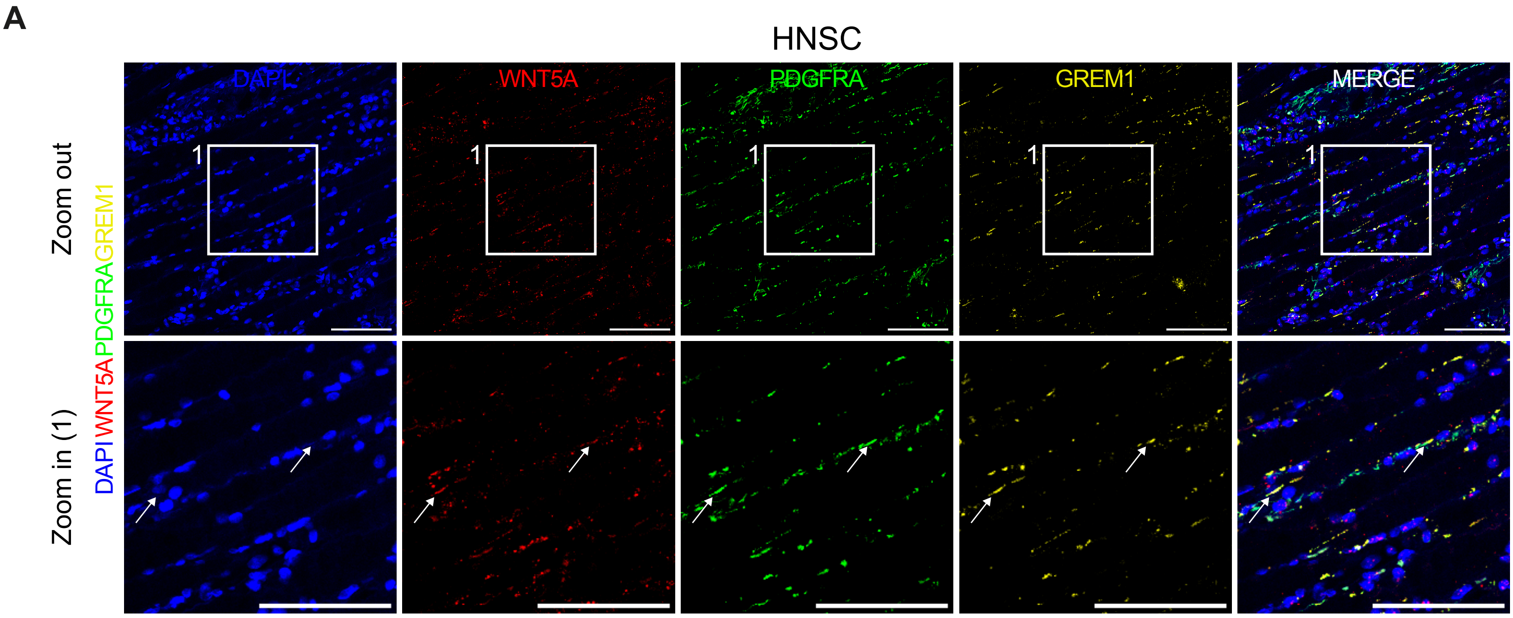
A
HNSC
DAPI
WNT5A
PDGFRA
GREM1
MERGE
Zoom out
Zoom in (1)
DAPI WNT5A PDGFRA GREM1
1

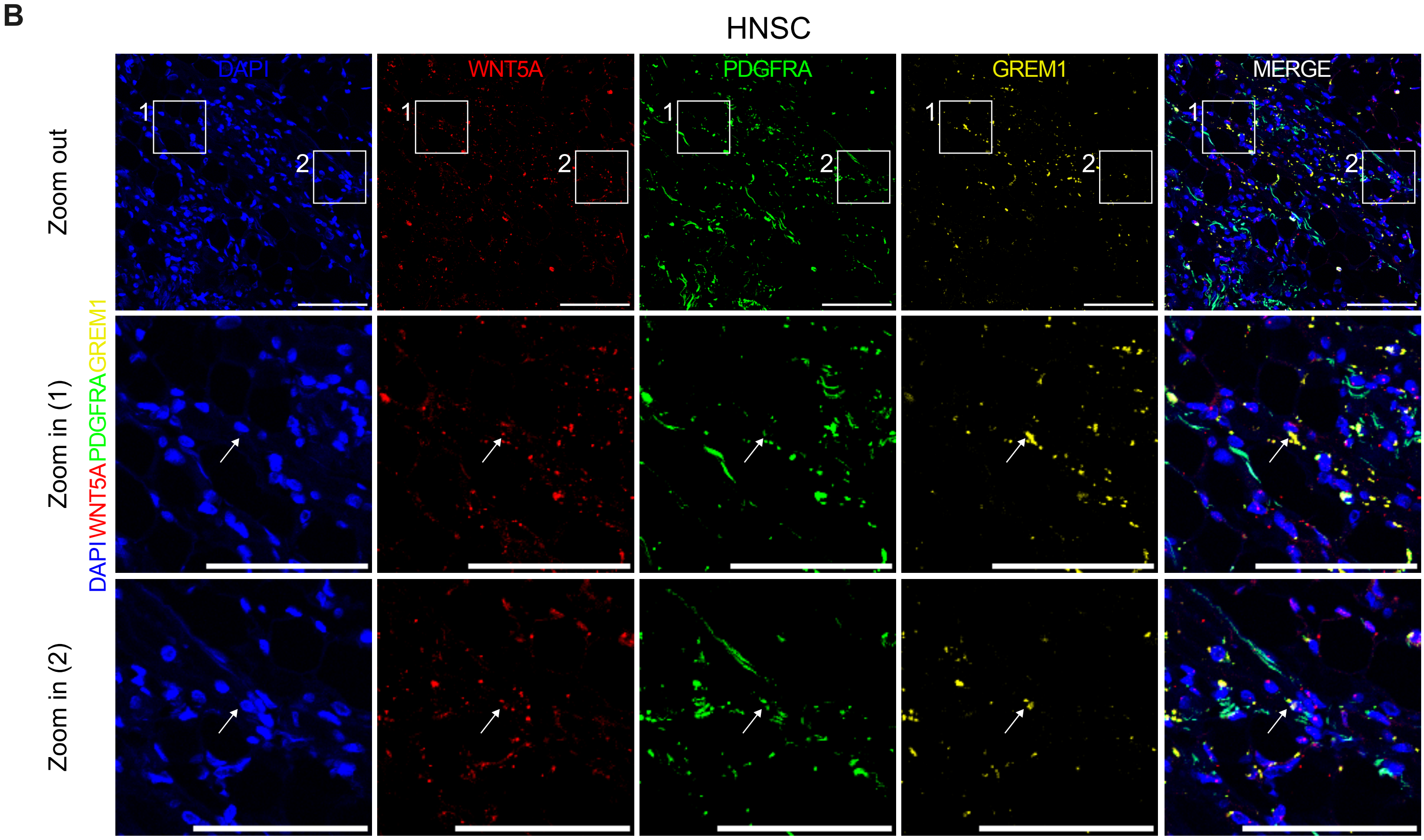
B
HNSC
DAPI
WNT5A
PDGFRA
GREM1
MERGE
Zoom out
Zoom in (1)
Zoom in (2)
DAPI WNT5A PDGFRA GREM1
1
2

Figure S15

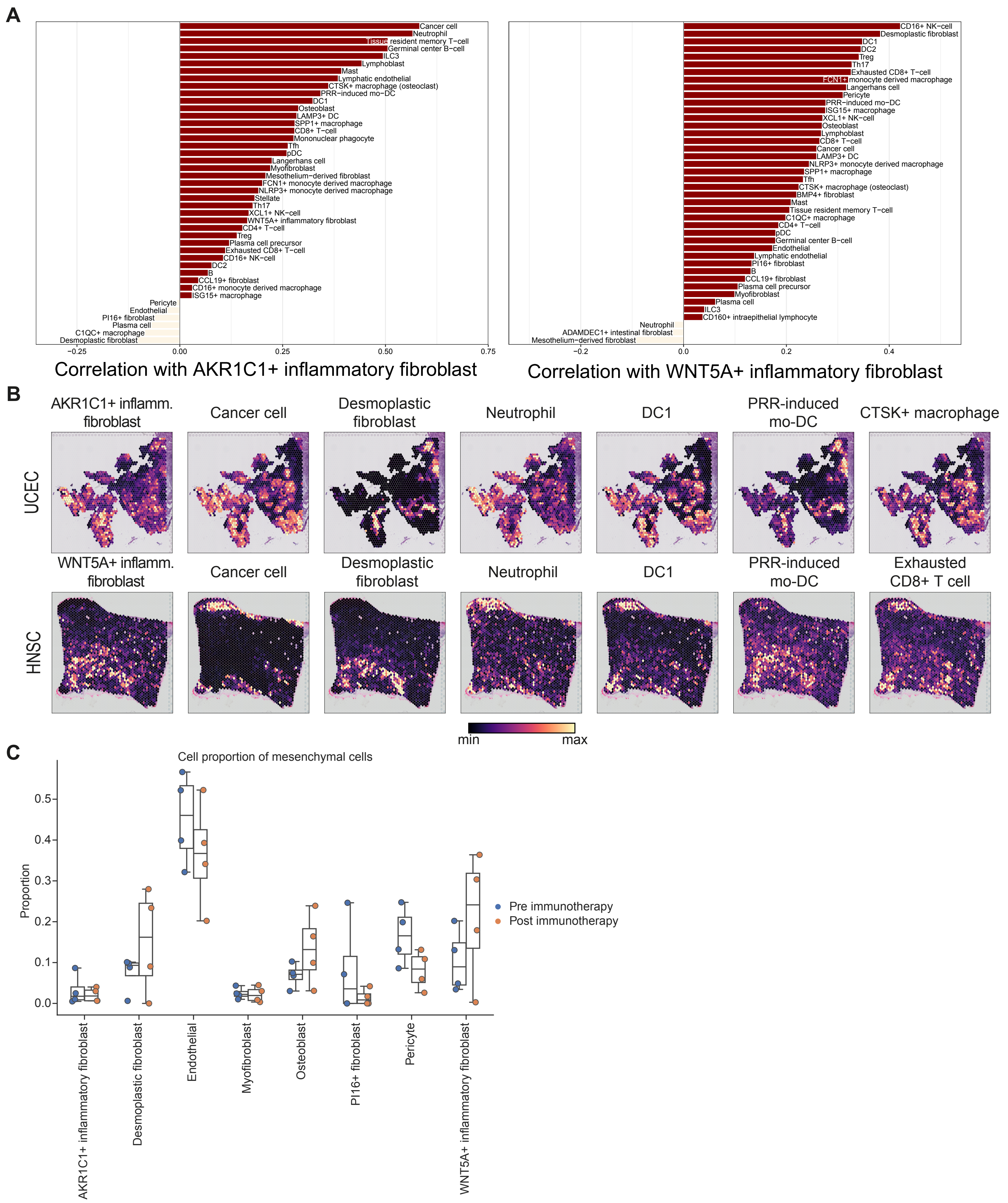
A
Cancer cell
Neutrophil
Tissue resident memory T−cell
Germinal center B−cell
ILC3
Lymphoblast
Mast
Lymphatic endothelial
CTSK+ macrophage (osteoclast)
PRR−induced mo−DC
DC1
Osteoblast
LAMP3+ DC
SPP1+ macrophage
CD8+ T−cell
Mononuclear phagocyte
Tfh
pDC
Langerhans cell
Myofibroblast
Mesothelium−derived fibroblast
FCN1+ monocyte derived macrophage
NLRP3+ monocyte derived macrophage
Stellate
Th17
XCL1+ NK−cell
WNT5A+ inflammatory fibroblast
CD4+ T−cell
Treg
Plasma cell precursor
Exhausted CD8+ T−cell
CD16+ NK−cell
DC2
B
CCL19+ fibroblast
CD16+ monocyte derived macrophage
ISG15+ macrophage
Pericyte
Endothelial
PI16+ fibroblast
Plasma cell
C1QC+ macrophage
Desmoplastic fibroblast
−0.25
0.00
0.25
0.50
0.75
Correlation with AKR1C1+ inflammatory fibroblast
CD16+ NK−cell
Desmoplastic fibroblast
DC1
DC2
Treg
Th17
Exhausted CD8+ T−cell
FCN1+ monocyte derived macrophage
Langerhans cell
Pericyte
PRR−induced mo−DC
ISG15+ macrophage
XCL1+ NK−cell
Osteoblast
Lymphoblast
CD8+ T−cell
Cancer cell
LAMP3+ DC
NLRP3+ monocyte derived macrophage
SPP1+ macrophage
Tfh
CTSK+ macrophage (osteoclast)
BMP4+ fibroblast
Mast
Tissue resident memory T−cell
C1QC+ macrophage
CD4+ T−cell
pDC
Germinal center B−cell
Endothelial
Lymphatic endothelial
PI16+ fibroblast
B
CCL19+ fibroblast
Plasma cell precursor
Myofibroblast
Plasma cell
ILC3
CD160+ intraepithelial lymphocyte
Neutrophil
ADAMDEC1+ intestinal fibroblast
Mesothelium−derived fibroblast
−0.2
0.0
0.2
0.4
Correlation with WNT5A+ inflammatory fibroblast
B
AKR1C1+ inflamm. fibroblast
Cancer cell
Desmoplastic fibroblast
Neutrophil
DC1
PRR-induced mo-DC
CTSK+ macrophage
UCEC
WNT5A+ inflamm. fibroblast
Cancer cell
Desmoplastic fibroblast
Neutrophil
DC1
PRR-induced mo-DC
Exhausted CD8+ T cell
HNSC
min
max
C
Cell proportion of mesenchymal cells
Proportion
0.0
0.1
0.2
0.3
0.4
0.5
AKR1C1+ inflammatory fibroblast
Desmoplastic fibroblast
Endothelial
Myofibroblast
Osteoblast
PI16+ fibroblast
Pericyte
WNT5A+ inflammatory fibroblast
Pre immunotherapy
Post immunotherapy

Figure S16

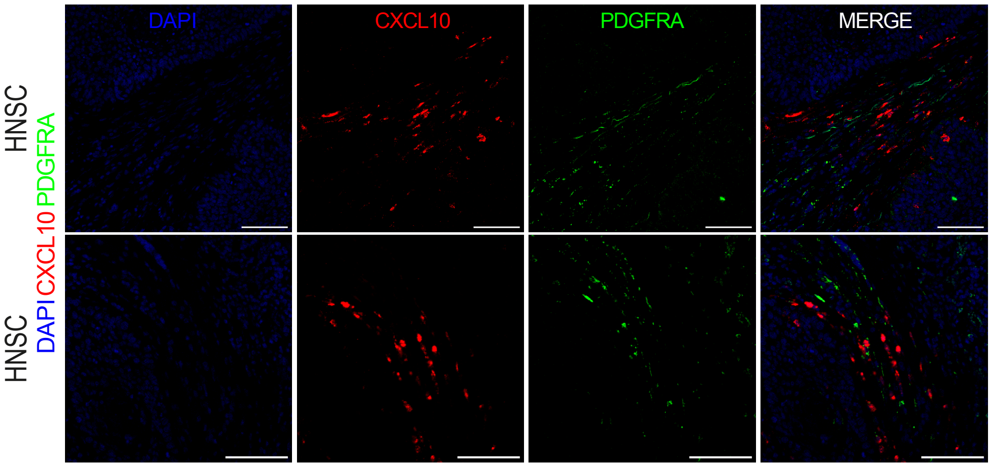

Figure S17

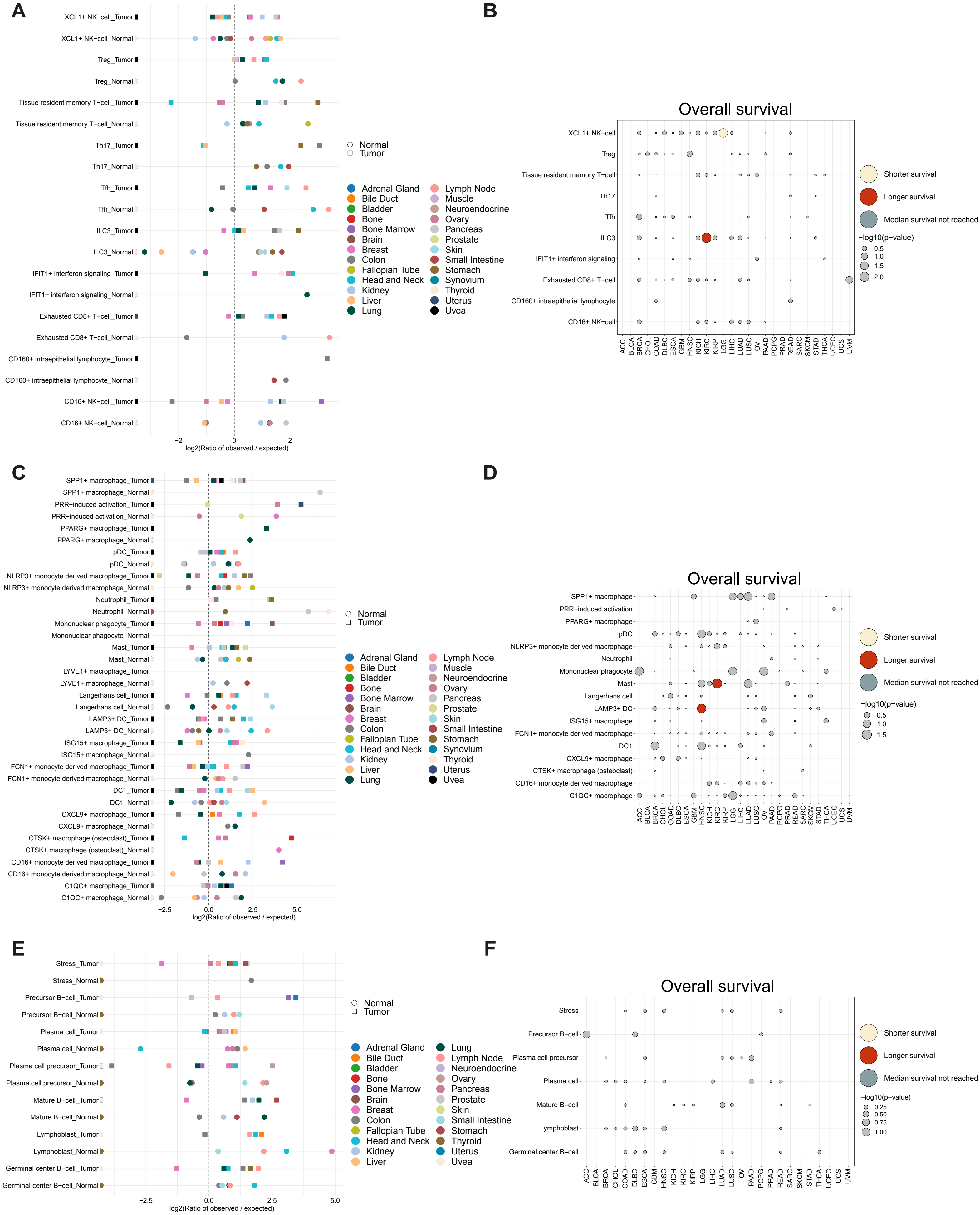

A
XCL1+ NK−cell_Tumor
XCL1+ NK−cell_Normal
Treg_Tumor
Treg_Normal
Tissue resident memory T−cell_Tumor
Tissue resident memory T−cell_Normal
Th17_Tumor
Th17_Normal
Tfh_Tumor
Tfh_Normal
ILC3_Tumor
ILC3_Normal
IFIT1+ interferon signaling_Tumor
IFIT1+ interferon signaling_Normal
Exhausted CD8+ T−cell_Tumor
Exhausted CD8+ T−cell_Normal
CD160+ intraepithelial lymphocyte_Tumor
CD160+ intraepithelial lymphocyte_Normal
CD16+ NK−cell_Tumor
CD16+ NK−cell_Normal
log2(Ratio of observed / expected)
Normal
Tumor
Adrenal Gland
Bile Duct
Bladder
Bone
Bone Marrow
Brain
Breast
Colon
Fallopian Tube
Head and Neck
Kidney
Liver
Lung
Lymph Node
Muscle
Neuroendocrine
Ovary
Pancreas
Prostate
Skin
Small Intestine
Stomach
Synovium
Thyroid
Uterus
Uvea
B
Overall survival
XCL1+ NK−cell
Treg
Tissue resident memory T−cell
Th17
Tfh
ILC3
IFIT1+ interferon signaling
Exhausted CD8+ T−cell
CD160+ intraepithelial lymphocyte
CD16+ NK−cell
ACC BLCA BRCA CHOL COAD DLBC ESCA GBM HNSC KICH KIRC KIRP LGG LIHC LUAD LUSC OV PAAD PCPG PRAD READ SARC SKCM STAD THCA UCEC UCS UVM
Shorter survival
Longer survival
Median survival not reached
−log10(p−value)
C
SPP1+ macrophage_Tumor
SPP1+ macrophage_Normal
PRR−induced activation_Tumor
PRR−induced activation_Normal
PPARG+ macrophage_Tumor
PPARG+ macrophage_Normal
pDC_Tumor
pDC_Normal
NLRP3+ monocyte derived macrophage_Tumor
NLRP3+ monocyte derived macrophage_Normal
Neutrophil_Tumor
Neutrophil_Normal
Mononuclear phagocyte_Tumor
Mononuclear phagocyte_Normal
Mast_Tumor
Mast_Normal
LYVE1+ macrophage_Tumor
LYVE1+ macrophage_Normal
Langerhans cell_Tumor
Langerhans cell_Normal
LAMP3+ DC_Tumor
LAMP3+ DC_Normal
ISG15+ macrophage_Tumor
ISG15+ macrophage_Normal
FCN1+ monocyte derived macrophage_Tumor
FCN1+ monocyte derived macrophage_Normal
DC1_Tumor
DC1_Normal
CXCL9+ macrophage_Tumor
CXCL9+ macrophage_Normal
CTSK+ macrophage (osteoclast)_Tumor
CTSK+ macrophage (osteoclast)_Normal
CD16+ monocyte derived macrophage_Tumor
CD16+ monocyte derived macrophage_Normal
C1QC+ macrophage_Tumor
C1QC+ macrophage_Normal
log2(Ratio of observed / expected)
D
Overall survival
SPP1+ macrophage
PRR−induced activation
PPARG+ macrophage
pDC
NLRP3+ monocyte derived macrophage
Neutrophil
Mononuclear phagocyte
Mast
Langerhans cell
LAMP3+ DC
ISG15+ macrophage
FCN1+ monocyte derived macrophage
DC1
CXCL9+ macrophage
CTSK+ macrophage (osteoclast)
CD16+ monocyte derived macrophage
C1QC+ macrophage
E
Stress_Tumor
Stress_Normal
Precursor B−cell_Tumor
Precursor B−cell_Normal
Plasma cell_Tumor
Plasma cell_Normal
Plasma cell precursor_Tumor
Plasma cell precursor_Normal
Mature B−cell_Tumor
Mature B−cell_Normal
Lymphoblast_Tumor
Lymphoblast_Normal
Germinal center B−cell_Tumor
Germinal center B−cell_Normal
log2(Ratio of observed / expected)
F
Overall survival
Stress
Precursor B−cell
Plasma cell precursor
Plasma cell
Mature B−cell
Lymphoblast
Germinal center B−cell

Figure S18

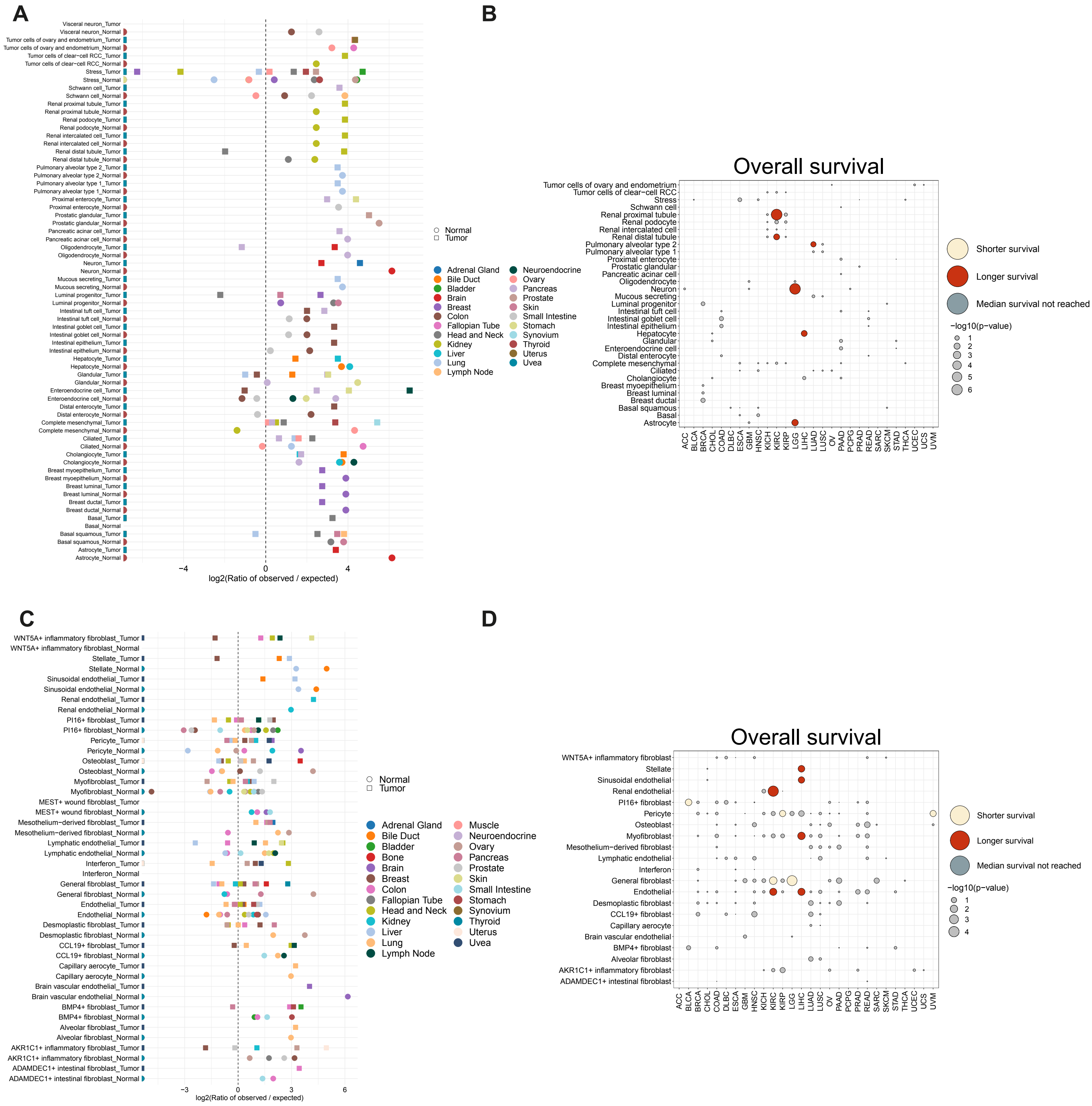

A
log2(Ratio of observed / expected)
Normal
Tumor
Adrenal Gland
Bile Duct
Bladder
Brain
Breast
Colon
Fallopian Tube
Head and Neck
Kidney
Liver
Lung
Lymph Node
Neuroendocrine
Ovary
Pancreas
Prostate
Skin
Small Intestine
Stomach
Synovium
Thyroid
Uterus
Uvea
B
Overall survival
Shorter survival
Longer survival
Median survival not reached
−log10(p−value)
C
log2(Ratio of observed / expected)
Normal
Tumor
Adrenal Gland
Bile Duct
Bladder
Bone
Brain
Breast
Colon
Fallopian Tube
Head and Neck
Kidney
Liver
Lung
Lymph Node
Muscle
Neuroendocrine
Ovary
Pancreas
Prostate
Skin
Small Intestine
Stomach
Synovium
Thyroid
Uterus
Uvea
D
Overall survival
Shorter survival
Longer survival
Median survival not reached
−log10(p−value)

Figure S19

A

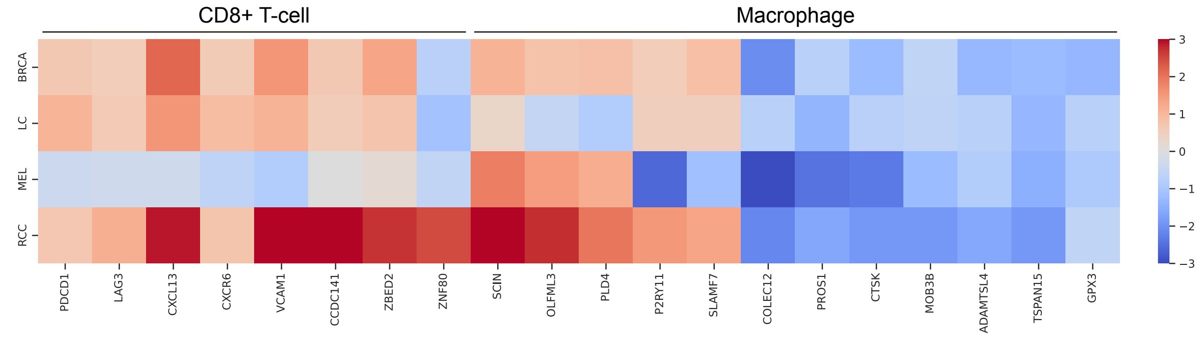

B

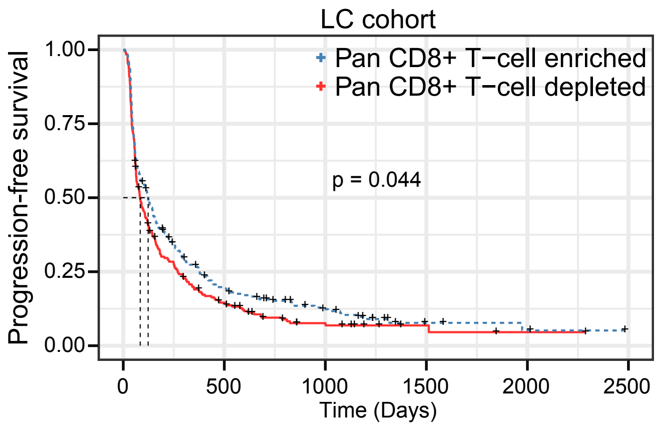

Figure S20

A

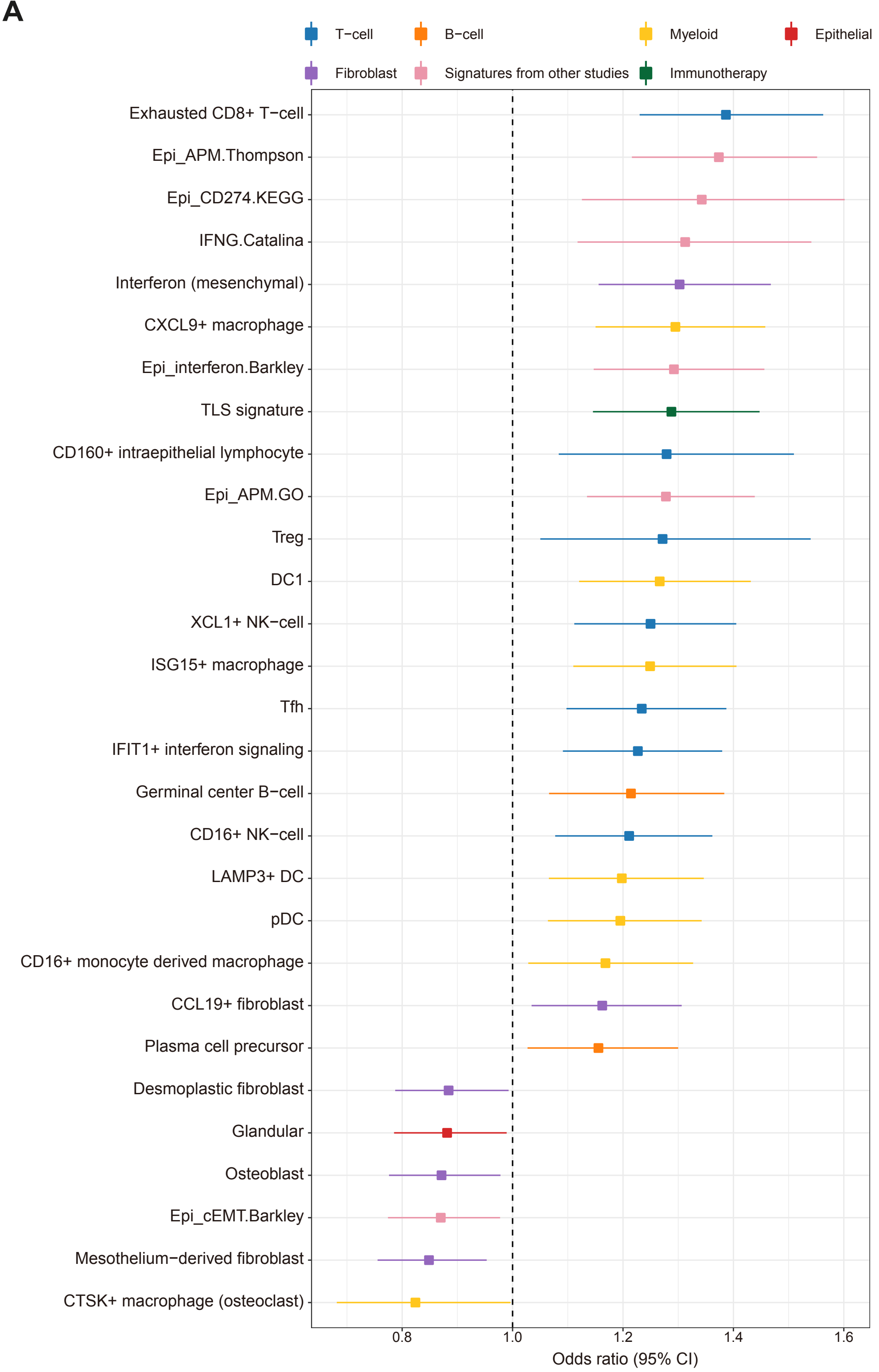

B

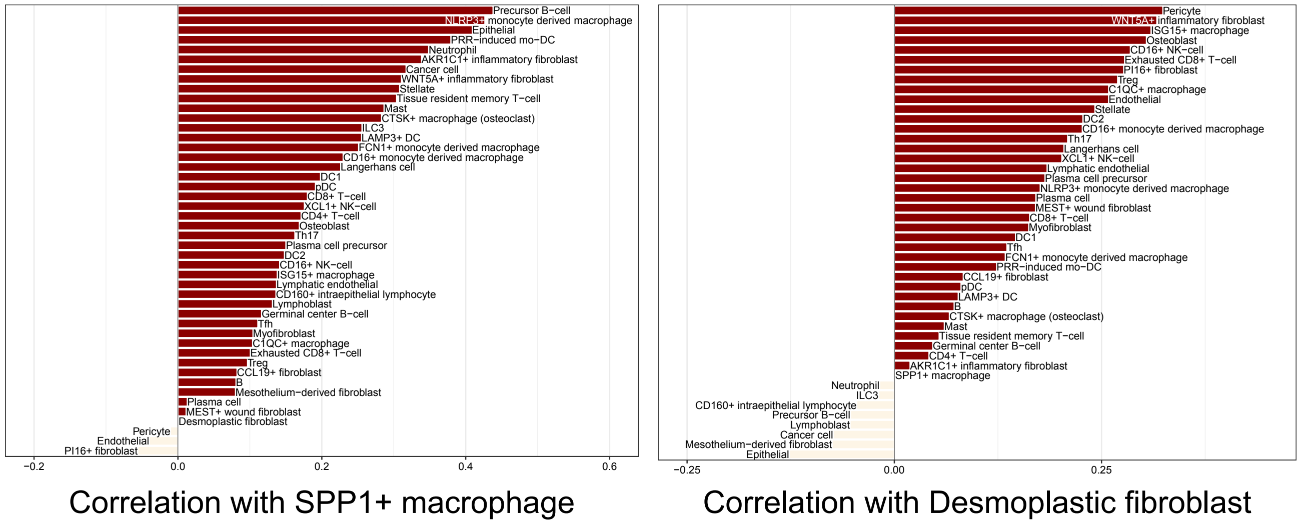

Supplement: Supplementary file 2 — Updated Supplementary Information [file 41467_2025_58068_MOESM2_ESM.pdf]
